# Beam-Specific Spot Guidance and Optimization for PBS Proton Treatment of Bilateral Head and Neck Cancers

**DOI:** 10.14338/IJPT-20-00060.1

**Published:** 2021-06-25

**Authors:** Karla Leach, Shikui Tang, Jared Sturgeon, Andrew K. Lee, Ryan Grover, Parag Sanghvi, James Urbanic, Chang Chang

**Affiliations:** 1 California Protons Cancer Therapy Center, San Diego, CA, USA; 2 Texas Center for Proton Therapy, Irving, TX, USA; 3 Department of Radiation Medicine and Applied Sciences, University of California, San Diego, CA, USA

**Keywords:** proton therapy, head and neck cancer, PBS, MFO, beam-specific spot placement guidance

## Abstract

**Purpose:**

A multi-field optimization (MFO) technique that uses beam-specific spot placement volumes (SPVs) and spot avoidance volumes (SAVs) is introduced for bilateral head and neck (H&N) cancers. These beam-specific volumes are used to guide the optimizer to consistently achieve optimal organ-at-risk (OAR) sparing with target coverage and plan robustness.

**Materials and Methods:**

Implementation of this technique using a 4-beam, 5-beam, and variant 5-beam arrangement is discussed. The generation of beam-specific SPVs and SAVs derived from target and OARs are shown. The SPVs for select fields are further partitioned into optimization volumes for uniform dose distributions that resemble those of single-field optimization (SFO). A conventional MFO plan that does not use beam-specific spot placement guidance (MFOcon) and an MFO plan that uses only beam-specific SPV (MFOspv) are compared with current technique (MFOspv/sav), using both simulated scenarios and forward-calculated plans on weekly verification computed tomography (VFCT) scans.

**Results:**

Dose distribution characteristics of the 4-beam, 5-beam, and variant 5-beam technique are demonstrated with discussion on OAR sparing. When comparing the MFOcon, MFOspv, and MFOspv/sav, the MFOspv/sav is shown to have superior OAR sparing in 9 of the 14 OARs examined. It also shows clinical plan robustness when evaluated by using both simulated uncertainty scenarios and forward-calculated weekly VFCTs throughout the 7-week treatment course.

**Conclusion:**

The MFOspv/sav technique is a systematic approach using SPVs and SAVs to guide the optimizer to consistently reach desired OAR dose values and plan robustness.

## Introduction

Cancers of the head and neck (H&N) present unique challenges in radiation therapy. A typical H&N target is surrounded by critical organs at risk (OARs) such as the oral cavity, parotids, larynx, and spinal cord. Proton therapy has the potential to spare these surrounding OARs by exploiting the characteristics of Bragg peaks, within which most of the radiation energy is deposited and no exit dose beyond [1–4]. To best use this intrinsic property of proton radiation for patient treatments, appropriate planning techniques must be used [5–8].

Recent advancement in proton delivery techniques has enabled active spot scanning, referred to as the *pencil beam scanning (PBS) modality*, to be used routinely in the clinic [9, 10]. Proton PBS treatment planning is an optimization process that puts together numerous proton spots of various energies at locations with proper weights. The conversion accuracy from Hounsfield units (HU) to proton relative stopping power determines the accuracy of where the proton path will end and consequently accuracy in proton dose calculation. This property, referred to as the *proton range uncertainty*, together with the uncertainties in patient setup, must be taken into account in the planning process. A robust optimization, which takes into account the uncertainties in patient setup and proton ranges, is therefore required for proton PBS treatment planning [11–13].

Different planning techniques have varying effects on not only the dosimetric outcomes but also the resultant plan's robustness against the uncertainties [14, 15]. Plans with independently optimized beams (single-field optimization, SFO), where each field contributes a uniform dose over the target, are in general more resilient to errors in patient setup and HU calibration. However, SFO plans are not able to use compensating dose distributions from more than 1 beam to spare OARs. On the other hand, plans that are optimized by simultaneously incorporating contributions from multiple beams (multi-field optimization, MFO) are typically more capable of achieving competing target and OAR dose objectives [16–22]. In addition, recent advancements in robust optimization have enabled MFO plans with improved robustness by incorporating setup and range uncertainties into the optimization process [23], and as a result greatly expanded the use of MFO in the clinics.

Proton PBS treatment plans using MFO have shown tremendous potential for H&N cancers [24–29]. However, the treatment planning system (TPS) optimizer relies heavily on user's judgements and inputs, which can create inconsistencies in plan quality. In this study, we explore an MFO technique that uses beam-specific spot placement volumes (SPVs) and spot avoidance volumes (SAVs), as well as SFO optimization structures within these spot guidance volumes, to guide the optimizer to find the solution that will consistently achieve optimal OAR sparing while maintaining the desired target coverage and plan robustness. We have also presented 3 variations of this planning technique by using 1 case in each variation and discussed the circumstances that make these variations most beneficial. Robustness of this planning technique was evaluated on 1 clinical case by using both simulated scenarios on the original planning computed tomography (CT), and forward-calculated original plans on the patient's subsequent weekly verification CT (VFCT) scans throughout the treatment course.

## Materials and Methods

All plans are optimized with robust minimum dose objectives set to the clinical target volumes (CTVs) for each prescription level. Identical robustness optimization settings, that is, 4% range uncertainty and 3-mm setup uncertainty, are used for all plans. Interbeam robust optimization was not used owing to prolonged planning time. This study was reviewed by the authors' institutional research infrastructure and was determined to be exempt from institutional review board approval.

### Spot Placement Volume/Spot Avoidance Volume Planning Technique: 4-Beam Arrangement

The first case examined is a squamous cell carcinoma of the left soft palate stage T2N2cM0 treated to 3 dose levels 70/63/56 GyRBE in 35 fractions. A case with this type of volume, which has no separation at midline in the oral cavity region, uses a 4-field arrangement: anterior-posterior (AP), posterior-anterior (PA), left anterior oblique (LAO), and right anterior oblique (RAO). Gantry angles close to ±60° and couch rotations of +15°/−15° are often associated with the RAO and LAO beams, respectively, to assist OAR sparing and to avoid the shoulder. A ranger shifter is often needed owing to shallow tumor depth and the low-energy limitations of the treatment delivery system.

Beam-specific SPVs and SAVs are used to guide the optimizer in the placement of proton spots to achieve desired OAR sparing and plan robustness. The SPV is derived from the planning target volume (PTV), which in turn is obtained from the CTV with an isotropic expansion based on setup uncertainty. For H&N cancers, a margin of 3 mm is typically used. The SPV is used to delimit the largest extent of each beam's spot placement. The exact location of proton spots is determined during optimization by the TPS. Each beam's SAV is derived from the OARs with typically 3-mm margin and adjusted on the basis of beam angle and proximity to the target. These 2 volumes, SPV and SAV, are synergistically used to guide beam-specific spot placements.

**Figure 1.**
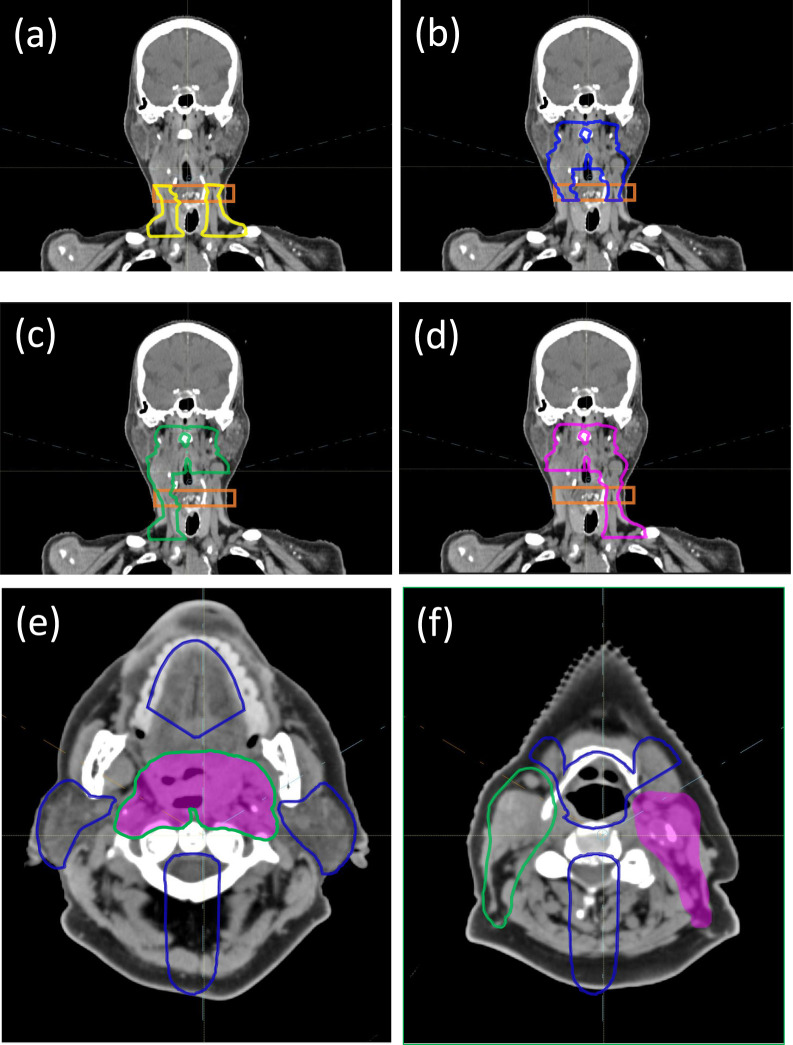
View of (a) AP, (b) PA, (c) RAO, and (d) LAO spot placement volumes (yellow, blue, green, and pink, respectively) with the transition region in orange. The SPV for LAO is in pink shade and RAO in green outline (e, f). Both RAO and LAO share the same SAV (in blue outline). The SPVs for both RAO and LAO are connected (e) across the midline and separated (f) below the parotids. Abbreviations: AP, anterior-posterior; LAO, left anterior oblique; PA, posterior-anterior; RAO, right anterior oblique; SAV, spot avoidance volume; SPV, spot placement volume.

As seen in **Figure 1a**, the SPV for the AP beam includes only the lower neck region, and its superior border must be at least 1.5 cm below the chin, excluding the oral cavity and avoiding the uncertainty in chin position reproducibility. The SPV for the PA beam is superior to the AP beam's SPV and its inferior border must overlap with the AP beam's SPV (**Figure 1b**) by at least 2 cm. This 2-cm overlap is slightly larger than the lateral penumbra of proton beams at this shallow depth, and it therefore allows the smooth dose gradients of the AP and PA beams to intersect inside this “transition” region (orange in Figure 1). The 2 anterior oblique beams with minor couch kicks, LAO and RAO, as seen in **Figure 1c** and **1d**, are typically used to cover the entire superior-inferior length of the target, but with sections on their respective contralateral side cropped for better OAR sparing. For example, the RAO beam's SPV ends on the left side where the PTV bifurcates inferiorly to the left parotid (**Figure 1c**), and vice versa for the RAO (**Figure 1d**).

Spot avoidance volumes are used to ensure that proton spots do not traverse, or stop in front of an OAR. The SAVs used by the LAO and RAO beams restrict spot placement around larynx, parotids, oral cavity, submandibular glands, cochlea, brainstem, and the spinal cord. Specifically, the SAVs are generated by expanding the OARs with a 3-mm expansion, taking a Boolean union of the expansions, and then subtracting the SPV with a 3-mm margin. Depending on the relative anatomy, additional manual editing of the SAV may be required to balance target coverage and OAR sparing. As an example, the SAV for the LAO and RAO beams in a 4-beam plan is shown in **Figure 1e** and **1f**. Here both LAO and RAO share the same SAV and it is edited around the oral cavity and submandibular glands to ensure that the medial section of the target is accessible by both beams. In addition, any metal dental fillings will also be included into the SAV so that no proton spots can be placed inside or through the metal. Note that here the spinal cord has an additional 5-cm posterior expansion to ensure that the anterior oblique beams do not place spots across the midline from the space posterior to the cord. This arrangement still allows the anterior oblique beams to deliver dose across the midline but only through the space anterior to the cord, that is, only by traversing inside the target. The SAV for the PA beam overlaps with the SAV of the LAO and RAO (shown in **Figure 1e** and **1f**) but does not include the cord portion because the PA beam needs access to the medial target. For TPSs that do not provide spot avoidance function, a manual process is needed to project the SAV along the beam path to be subtracted from the SPV. Additional lateral margins, up to 5 to 8 mm, to the SAV may be required to achieve the same level of OAR sparing owing to lateral spot margins.

In addition to the spot placement guidance above, further segmented optimization structures within the SPVs are needed for the optimization of SFO-like dose distributions. Two SFO structures are created for the PA and AP beam at the superior and inferior neck. The dosimetric goal is for the PA beam to provide half of the prescription in the superior neck with the remaining half delivered in MFO distributions by the anterior obliques. While in the inferior neck, the AP will deliver half of the prescription and the 2 anterior obliques provide the other half to their corresponding ipsilateral side of the target. Note that the transition region between the AP-PA beams is specifically left out of these SFO structures to allow the AP and PA beams to fade toward the superior and inferior directions, respectively. This dose distribution emulates that of a craniospinal irradiation (CSI) and provides a smooth dose gradient into the transition region, thus alleviating the potential dose heterogeneity due to various uncertainties [30].

### SPV/SAV Planning Technique: 5-Beam Arrangement

**Figure 2.**
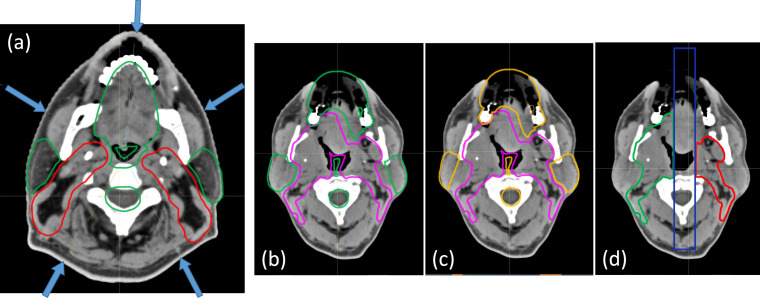
(a) Beam arrangement for the 5-beam techniques. SAVs are shown in green. (b) SPV (pink) and SAV (green) for the LPO. (c) SPV (pink) and SAV (orange) for the RPO beam. (d) SFO sections of RPO and LPO are separated by a 2-cm gap at midline. The SFO section of the RPO (green) and the SFO section of the LPO (red) are seen to be separated by a 2-cm control region (blue). Abbreviations: LPO, left posterior oblique; RPO, right posterior oblique; SAV, spot avoidance volume; SFO, single-field optimization; SPV, spot placement volume.

The second case examined is a sinonasal undifferentiated carcinoma T2N0M0 treated with 2 dose levels 63/56 GyRBE in 35 fractions. A case with this type of volume where the target is separated at midline around the oral cavity is best suited for the 5-beam technique. This technique is identical to the 4-beam in the lower neck region in that no posterior beams are used for the inferior neck nodes. Difference exists superiorly where the single PA is replaced by 2 posterior obliques. As seen in **Figure 2a**, the posterior oblique beam angles are chosen to be parallel to the interface between the ipsilateral targets and parotid. Owing to the target separation into distinct left and right sections, the anterior oblique beams' SPVs are defined such that no proton spots are placed across midline. This arrangement provides optimal sparing of the various OARs situated along the medial section, such as the oral cavity and pharyngeal constrictor. Without the PA beam, the posterior obliques require individual SFO structures to deliver half the prescribed dose to their respective ipsilateral side targets, while the remaining half will be delivered in MFO distributions by ipsilateral anterior oblique and contralateral posterior oblique.

### Spot Placement Volume/Spot Avoidance Volume Planning Technique: Variant 5-Beam Arrangement

The third case examined is a squamous cell carcinoma of the right base of tongue stage T4N2bM0 stage III treated to 3 dose levels 70/63/56 GyRBE in 35 fractions. This treatment volume is similar to the first case (treated with the 4-field arrangement) in that it is connected at midline in the oral cavity region. However, portions of the target are surrounded by metal dental fillings that prevent access by the anterior beams. The 4-beam arrangement is therefore not applicable, since both posterior obliques are needed to capture targets posterior to the metal dental fillings and we do not use a single beam to deliver full prescription to any part of the target. A variant 5-beam technique is therefore used where all 4 oblique beams can place spots across the midline unless otherwise blocked by their respective SAVs. An SFO section on the ipsilateral side is defined within each of the posterior oblique beam's SPV, as seen in **Figure 2b** to **2d**. The dosimetric goal is again to have the posterior oblique beams deliver half of the prescribed dose uniformly in a SFO-like manner to the ipsilateral side of the target, and the other half of the prescription delivered as MFO from the other 3 oblique beams to achieve desired OAR sparing. Also seen in **Figure 2d** is a 2-cm-wide “control” region (blue) separating the 2 SFO sections (green and red) of the posterior oblique beams. Like the transition region (orange) in **Figure 1a** to **1d**, this control region permits proper dose gradients for the 2 uniform dose distributions from the SFO sections and avoids dose heterogeneity at the junction due to setup, range, and anatomic uncertainties.

### Dosimetric and Robustness Evaluations

For comparison, the second bilateral H&N case with sinus involvement was re-planned by using 2 additional MFO techniques: conventional MFO (MFOcon) without any SPV or SAV volumes and MFO with only SPV volumes (MFOspv). These additional MFO techniques are commonly used in PBS treatment planning for H&N cancers. All plans used the same 5 beams and robustness settings. The optimization objectives on the original target and OAR contours are identical between the MFOcon, MFOspv, and MFOspv/sav plans. Detailed planning objectives are included in **Supplemental Table S1**. All 3 MFO plans are normalized such that 97% of the CTV63 volume is covered by prescription, that is, D97% = 63 GyRBE. These 3 plans are then designated as the nominal plans in Tables 1 and 2.

**Table 1.** Robustness evaluation of the 3 MFO planning techniques using simulated scenarios.

	**CTV63, D97%**	**CTV56, D97%**	**Cord, Dmax**	**Chiasm, D0.05cc**	**Brainstem, Dmax**	**Parotid left, Dmean**	**Parotid right, Dmean**	**Lacrimal gland left, Dmean**	**Lacrimal gland right, Dmean**	**Submandibular gland left, Dmean**	**Larynx, Dmean**	**Oral cavity, Dmean**	**Cochlea left, Dmax**	**Cochlea right, Dmax**	**Temporal lobe left, D2.0cc**	**Temporal lobe right, D2.0cc**	**External, D1.0cc**
MFO using SPV and SAV																	
Nominal	63.2	56.4	35.6	46.3	45.5	11.8	12.6	17.0	16.8	29.1	14.6	9.2	14.4	16.1	42.5	42.1	67.4
Scenario 1	60.6	54.4	41.2	41.0	40.8	9.6	17.0	18.6	**27.1**	36.0	19.7	13.0	10.5	11.3	34.8	45.8	73.2
Scenario 2	61.6	54.7	39.5	51.8	47.7	11.8	20.3	14.7	19.2	34.5	18.4	9.1	16.9	26.1	39.6	53.8	72.7
Scenario 3	61.0	52.6	35.9	45.4	54.4	9.6	16.5	14.6	25.3	29.4	18.1	11.5	13.4	15.7	38.5	45.7	72.9
Scenario 4	61.1	52.4	35.3	**55.1**	**61.9**	11.6	20.0	10.6	16.3	28.3	17.5	7.8	24.9	33.1	42.6	53.9	71.7
Scenario 5	60.0	54.6	34.9	39.7	41.0	16.3	10.8	25.9	18.2	42.9	19.9	15.1	10.0	10.5	42.8	33.2	73.1
Scenario 6	61.2	54.8	31.2	51.4	47.4	19.2	13.9	21.1	13.2	41.9	18.3	11.0	22.9	24.2	49.7	40.9	70.6
Scenario 7	58.9	53.6	35.4	43.6	50.9	16.2	10.5	22.1	17.5	36.3	19.1	13.3	13.7	13.5	46.5	34.8	73.6
Scenario 8	58.9	53.6	34.0	53.5	**57.7**	19.1	13.4	17.4	10.9	35.1	18.1	9.5	29.2	30.3	52.5	42.4	71.9
Scenario 9	59.1	52.3	42.7	38.9	35.9	7.3	12.9	17.2	24.1	22.7	13.8	10.2	10.2	8.8	34.0	40.7	70.1
Scenario 10	59.5	53.4	40.2	50.7	40.2	9.2	15.9	13.4	17.0	21.1	11.9	6.3	14.2	17.0	38.8	49.4	69.6
Scenario 11	59.9	51.5	36.8	42.8	46.2	6.7	12.3	13.0	22.0	16.3	11.9	8.7	12.6	12.3	38.2	41.2	69.7
Scenario 12	58.8	51.9	36.4	53.3	52.2	8.2	15.4	9.1	14.0	14.9	10.8	5.2	22.2	23.8	42.2	49.7	69.3
Scenario 13	57.7	53.6	36.4	35.0	34.7	13.0	8.0	23.4	16.2	31.0	14.5	11.7	9.0	9.1	40.7	30.5	69.7
Scenario 14	58.7	54.8	33.0	49.8	39.9	15.4	10.8	18.5	11.6	29.5	12.9	7.5	18.5	15.8	48.0	37.5	69.1
Scenario 15	56.7	52.9	36.9	38.6	41.3	12.8	7.2	19.4	15.7	24.7	13.7	10.1	12.8	10.8	44.8	32.6	69.2
Scenario 16	56.2	53.9	34.7	50.9	50.3	15.0	9.7	14.7	9.4	23.1	12.5	6.3	26.0	22.2	50.9	39.3	69.7
MFO using SPV only																	
Nominal	63.3	56.2	31.6	46.5	53.2	20.6	21.3	21.7	21.0	28.0	20.6	13.9	17.4	19.5	36.9	40.3	67.1
Scenario 1	62.4	54.7	30.7	43.5	45.9	16.9	24.3	19.1	**26.3**	37.4	25.6	18.3	11.7	16.3	28.3	40.8	71.1
Scenario 2	62.3	54.5	30.7	**54.**1	53.3	20.6	**27.7**	20.0	25.7	36.3	22.9	13.9	18.2	25.6	35.7	52.5	70.1
Scenario 3	61.7	53.2	32.9	47.7	55.0	16.4	24.1	19.0	**27.2**	30.7	22.1	15.8	13.7	18.7	31.7	43.1	70.4
Scenario 4	61.9	52.1	32.7	**56.0**	**62.5**	19.9	**27.3**	19.0	24.5	29.7	19.9	11.7	22.6	29.9	38.5	53.9	69.2
Scenario 5	60.6	54.8	30.2	41.2	43.5	23.0	18.5	**26.8**	17.4	39.6	27.0	19.8	13.5	13.2	38.3	29.8	70.0
Scenario 6	61.3	55.1	29.5	52.8	54.3	**27.2**	22.0	25.5	19.0	38.5	24.0	15.4	22.8	20.2	46.6	40.4	69.5
Scenario 7	60.1	53.3	32.8	48.1	53.5	22.5	18.4	**26.2**	19.6	32.1	24.2	17.3	16.3	14.7	42.5	31.8	69.6
Scenario 8	60.0	52.8	30.9	**56.1**	**62.9**	**26.4**	21.4	24.8	19.5	31.1	21.5	13.2	28.1	24.9	49.8	41.7	68.5
Scenario 9	61.3	53.0	31.6	41.2	43.2	15.7	21.7	17.4	22.4	26.1	21.9	15.5	11.4	16.0	24.4	36.2	69.1
Scenario 10	60.6	53.3	32.0	52.0	50.4	19.4	**25.3**	18.2	22.9	24.9	18.9	11.2	16.1	23.6	31.2	48.3	68.6
Scenario 11	59.9	52.8	33.7	43.0	50.3	15.0	21.4	17.3	23.3	18.9	18.4	13.3	13.8	18.7	28.2	38.4	68.8
Scenario 12	59.9	53.2	33.7	53.2	**57.5**	18.3	24.7	17.3	21.6	17.7	15.9	9.3	21.0	28.4	34.1	49.0	68.2
Scenario 13	59.1	54.3	29.9	37.0	38.1	21.5	16.6	23.9	15.0	27.8	23.6	16.9	13.2	12.8	34.0	25.7	68.2
Scenario 14	59.0	55.0	29.7	48.4	50.1	**25.5**	20.2	22.6	17.2	26.5	20.5	12.5	20.7	18.8	41.9	36.0	68.4
Scenario 15	58.6	53.0	32.1	39.8	47.5	21.1	16.1	23.4	17.4	20.4	20.8	14.7	16.1	14.4	38.0	28.2	67.9
Scenario 16	58.3	54.2	30.8	49.0	**58.3**	24.7	19.3	21.7	17.6	19.2	18.0	10.7	26.2	23.9	44.6	37.8	67.5
Conventional MFO																	
Nominal	63.3	56.5	32.4	45.0	50.5	21.0	21.7	25.1	22.1	28.3	20.7	21.5	16.2	18.4	36.7	41.3	68.5
Scenario 1	61.8	54.7	33.0	41.1	44.5	17.5	**25.6**	21.0	**26.1**	36.5	23.8	23.9	10.3	15.6	26.7	42.9	75.2
Scenario 2	62.0	54.9	32.5	**56.8**	51.6	22.0	**28.5**	24.1	25.5	35.2	22.8	21.1	17.4	23.4	35.0	53.7	76.2
Scenario 3	61.3	53.8	31.6	45.1	53.3	16.5	24.4	23.2	**28.0**	28.4	22.3	21.6	12.8	18.3	32.3	45.3	75.2
Scenario 4	61.6	53.9	31.3	**55.8**	**61.9**	20.8	**26.8**	25.7	25.5	26.9	21.3	19.0	23.2	29.3	39.5	54.5	74.7
Scenario 5	61.5	54.9	33.6	40.2	42.0	23.1	19.5	**27.8**	17.8	39.4	25.4	25.8	11.9	13.1	36.8	32.1	73.0
Scenario 6	61.3	55.5	32.6	53.6	50.9	**28.2**	22.0	**29.3**	19.1	38.7	24.1	23.1	21.8	19.0	46.2	41.9	73.0
Scenario 7	60.7	54.5	32.0	45.7	54.0	22.1	18.2	**28.5**	20.5	30.6	24.8	23.4	15.1	14.5	42.4	34.0	73.4
Scenario 8	60.4	55.1	31.6	**55.1**	**61.6**	**26.9**	20.4	**29.9**	20.6	29.6	23.2	20.9	27.3	24.2	50.5	43.2	71.4
Scenario 9	60.7	52.9	34.1	35.9	41.2	16.0	23.4	18.3	23.9	29.5	20.4	22.9	10.4	15.2	23.7	37.3	71.0
Scenario 10	59.7	52.6	33.3	50.5	47.0	20.2	**26.2**	21.8	23.6	27.9	19.2	20.1	16.3	21.2	31.1	48.6	71.9
Scenario 11	58.7	52.5	32.8	38.0	47.1	15.2	22.3	20.8	25.8	21.0	19.3	20.7	12.3	17.5	29.4	39.3	71.4
Scenario 12	58.8	52.6	32.2	49.3	56.1	19.0	24.6	23.7	23.9	19.2	17.9	18.2	21.8	27.5	36.0	49.0	69.8
Scenario 13	59.1	54.0	34.3	34.5	35.8	21.4	17.7	24.1	16.6	32.2	22.3	24.7	12.2	12.3	33.2	27.2	70.2
Scenario 14	58.2	55.1	33.8	48.6	46.5	**26.1**	20.1	**26.1**	18.2	31.1	20.8	22.0	20.9	17.5	41.4	36.8	70.2
Scenario 15	58.0	53.3	33.3	40.1	46.1	20.6	16.5	25.3	19.4	23.4	21.9	22.4	15.0	14.3	38.5	29.6	69.3
Scenario 16	57.6	54.5	32.3	51.4	55.8	25.0	18.5	**27.0**	19.7	22.1	20.1	19.9	26.7	23.0	45.2	38.5	68.8

**Abbreviations:** MFO, multi-field optimization; CTV, clinical target volume; SPV, spot placement volume; SAV, spot avoidance volume.

**Table 2.** Interfraction plan robustness evaluation of the 3 MFO planning techniques using VFCTs taken weekly over the treatment course.

	**CTV63, D97%**	**CTV56, D97%**	**Cord, Dmax**	**Chiasm, D0.05cc**	**Brainstem, Dmax**	**Parotid left, Dmean**	**Parotid right, Dmean**	**Lacrimal gland left, Dmean**	**Lacrimal gland right, Dmean**	**Submandibular gland left, Dmean**	**Larynx, Dmean**	**Oral cavity, Dmean**	**Cochlea left, Dmax**	**Cochlea right, Dmax**	**Temporal lobe left, D2.0cc**	**Temporal lobe right, D2.0cc**	**External, D1.0cc**
MFO using SPV and SAV																	
Nominal	63.2	56.4	35.6	46.3	45.5	11.8	12.6	17.0	16.8	29.1	14.6	9.2	8.2	8.2	42.5	42.1	67.4
VFCT 1	62.2	55.1	33.8	53.8	48.1	12.6	13.6	16.2	19.6	25.9	12.7	11.1	9.4	8.5	40.7	38.4	69.4
VFCT 2	62.8	56.2	32.6	**55.3**	46.3	14.0	12.0	16.2	17.8	30.3	14.2	9.7	8.5	8.5	41.2	39.8	68.0
VFCT 3	62.7	56.1	32.4	**55.3**	47.3	14.5	12.1	14.1	18.1	33.3	15.5	10.0	8.3	8.5	42.3	42.8	68.3
VFCT 4	62.2	56.0	35.4	53.5	42.9	11.8	13.5	18.7	18.0	31.5	16.9	10.6	8.3	8.9	41.2	42.8	68.3
VFCT 5	62.7	56.0	35.2	**54.4**	47.7	13.6	13.1	16.7	16.8	34.5	17.0	10.0	8.5	9.5	42.4	44.1	69.5
VFCT 6	62.5	55.8	32.7	**54.7**	45.1	14.0	12.9	16.1	16.1	35.3	18.3	12.4	8.8	10.6	42.5	45.3	69.4
VFCT 7	62.2	55.7	35.0	**56.6**	48.2	14.8	14.1	14.8	15.5	37.8	21.3	12.9	9.0	10.2	43.1	44.6	70.6
MFO using SPV only																	
Nominal	63.3	56.2	31.6	46.5	53.2	20.6	21.3	21.7	21.0	28.0	20.6	13.9	17.4	19.5	36.9	40.3	67.1
VFCT 1	62.4	54.9	31.0	**55.7**	52.9	20.9	22.0	20.6	21.0	29.2	19.4	15.6	12.0	12.2	33.9	37.0	68.1
VFCT 2	63.0	55.9	30.6	**58.6**	**54.2**	22.4	20.7	20.6	21.6	29.3	21.2	14.2	12.2	11.9	35.6	38.8	67.8
VFCT 3	63.3	55.9	30.6	**58.9**	**54.1**	22.8	20.7	19.8	23.0	30.6	22.7	14.6	12.2	11.6	37.1	41.8	67.8
VFCT 4	63.1	55.9	31.3	**55.3**	53.1	20.2	22.4	22.7	20.9	28.5	23.5	14.7	11.4	13.0	36.0	40.0	68.7
VFCT 5	63.3	56.0	31.2	**57.9**	51.5	21.9	21.6	22.2	21.2	31.1	24.0	14.3	12.0	12.5	38.1	41.8	68.3
VFCT 6	63.1	55.8	31.4	**57.6**	53.4	22.6	21.5	21.4	21.3	31.7	23.7	15.5	12.4	13.1	37.8	42.2	69.2
VFCT 7	62.8	55.7	32.4	**57.4**	**54.4**	22.2	22.1	21.8	21.6	33.9	27.6	16.0	11.9	12.2	38.3	41.1	69.0
Conventional MFO																	
Nominal	63.3	56.5	32.4	45.0	50.5	21.0	21.7	25.1	22.1	28.3	20.7	21.5	16.2	18.4	36.7	41.3	68.5
VFCT 1	62.2	54.9	32.3	53.4	49.9	21.2	22.8	23.3	22.6	29.6	19.7	22.3	11.3	11.9	34.8	38.1	71.2
VFCT 2	63.1	56.0	32.5	**61.4**	52.1	22.7	21.5	24.0	23.0	29.7	21.2	21.6	11.2	11.5	35.8	40.0	69.7
VFCT 3	63.1	56.0	32.5	**61.4**	51.9	23.5	21.4	24.3	24.0	30.7	22.1	21.7	11.2	11.4	36.9	43.4	70.5
VFCT 4	63.0	56.2	33.1	**61.8**	49.9	21.0	23.4	25.8	21.8	28.5	22.3	21.5	10.6	12.4	36.3	40.9	70.7
VFCT 5	63.2	56.0	32.4	**61.3**	50.4	22.5	22.3	26.6	22.3	30.7	23.3	21.5	11.0	12.1	37.7	43.1	71.3
VFCT 6	63.0	56.1	32.3	**60.4**	50.5	23.5	22.2	25.7	22.3	30.9	22.6	21.9	11.4	12.4	37.7	43.4	72.0
VFCT 7	62.7	56.0	32.3	**62.2**	52.3	22.5	22.6	27.5	22.9	32.3	25.8	21.9	10.8	11.8	38.8	42.5	72.7

**Abbreviations:** MFO, multi-field optimization; VFCT, verification computed tomography; CTV, clinical target volume; SPV, spot placement volume; SAV, spot avoidance volume.

Plan robustness for these 3 MFO plans were evaluated by using both simulated uncertainty scenarios and forward-calculated plans on VFCTs taken over the course of the patient's treatment. The robustness evaluation shown in Table 1 and **Supplemental Figure S1** is part of the standard physics check for all patients before treatment starts. For each uncertainty scenario shown in Table 1, robustness of these 3 MFO plans was evaluated by deliberately shifting the location of the isocenter by ±0.3 cm in the x, y, and z directions to simulate setup errors, together with ±4% density perturbation to account for range uncertainties. For example, scenario 1 corresponds to an isocenter shift of 3 mm to the right, 3 mm to the anterior, and 3 mm to the inferior, as well as a 4% overrange in HU-to-stopping power calibration. A total of 16 different scenarios are evaluated.

Weekly VFCTs were taken during the treatment course. These VFCTs were registered to the planning CT, and the various target and OAR structures were transferred to the VFCTs and reviewed by the attending physician. The 3 nominal MFO plans were then forward-calculated on these VFCTs for interfractional robustness evaluation. The results are summarized in Table 2 and **Supplemental Figure S2**. Values that do not meet our clinical criteria are highlighted in bold/yellow.

## Results

### The 4-Beam Arrangement

**Figure 3.**
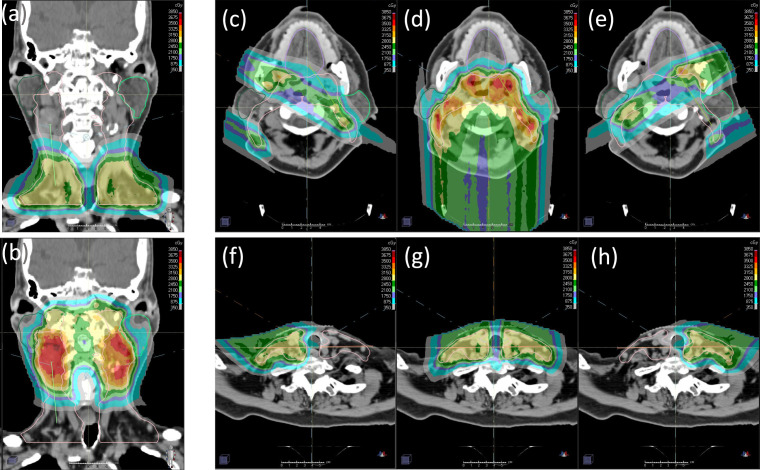
Dose distributions of the AP (a) and PA (b) beams (coronal), and the RAO (c, f), PA (b, d), and LAO (e, h) beams (axial) for the 4-beam technique. For the superior portion of the target (c, d, e), half of the prescription is delivered through the PA beam by using a uniform dose criterion. Only AP, RAO, and LAO are used for the inferior portion of the target (f, g, h). Abbreviations: AP, anterior-posterior; PA, posterior-anterior; LAO, left anterior oblique; RAO, right anterior oblique.

Dose distributions for the 4-beam technique are shown in Figure 3. As seen in **Figure 3a** and **3b**, the uniform dose objectives ensured that the AP and PA beams delivered a uniform, that is, SFO-like, dose distribution to their respective SFO structures within each SPV (excluding the transition region). Note that the PA beam's dose distribution shows 3 target dose levels (CTV56, CTV63, and CTV70), since this part of the target has simultaneous integrated boost. The AP beam on the other hand treats the inferior nodes and has only 1 target dose level (CTV56). Note that SFO structures must be created separately for each dose level. A uniform dose objective set to the overall PTV helps ensure overall dose homogeneity and guides the LAO and RAO beams to deliver the remaining half of the prescription, using the uniform dose distributions from AP and PA as a baseline. This results in the anterior oblique beams producing SFO-like dose distributions in the inferior neck when their SPVs overlap with the AP beam's SFO region; it also allows MFO dose distributions in the superior neck for better OAR sparing (**Figure 3c** to **3h**). This beam arrangement prevents dose spillage across the midline in the inferior neck and avoids entering the parotids in the superior neck for maximum OAR sparing.

### The 5-Beam Arrangements: Standard and Variant

**Figure 4.**
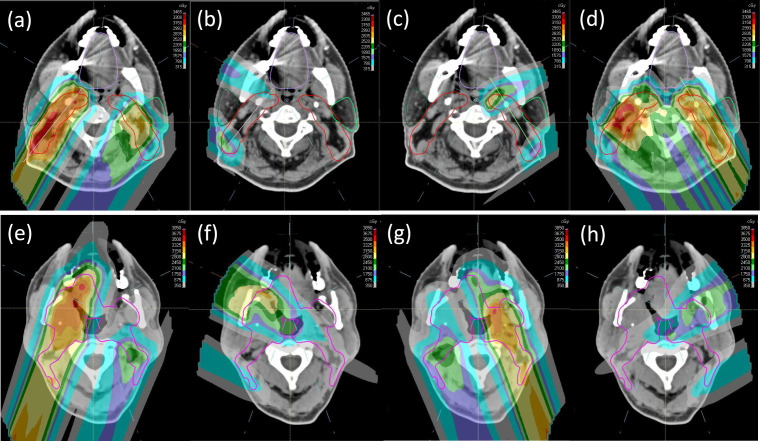
Dose distributions of (a) RPO, (b) RAO, (c) LAO, and (d) LPO beams for the 5-beam technique at levels where the target can be separated into left and right segments. Dose distributions from the (e) RPO, (f) RAO, (g) LPO, and (h) LAO beams for the variant 5-beam technique at levels where the target has medial involvement. Abbreviations: LAO, left anterior oblique; LPO, left posterior oblique; RAO, right anterior oblique; RPO, right posterior oblique.

Dose distributions for the 5-beam techniques are shown in Figure 4. Unlike the 4-beam technique, the posterior oblique beams in the 5-beam arrangement are also used to treat around the spinal cord to the contralateral side behind the contralateral parotids. The posterior oblique beams' SAVs do not include a posterior extension of the spinal cord (and brainstem if applicable). As demonstrated in **Figure 4a** to **4d** using standard 5-beam technique, at levels where the target volume can be separated into disjoint left and right segments, the segment on the left receives half of the prescription uniformly (ie, SFO-like) from the left posterior oblique (LPO) beam, and the other half of the prescribed dose is contributed by an MFO combination from the right posterior oblique (RPO) and LAO beams. The contralateral anterior oblique beam, that is, RAO, does not contribute to the target on the left in this case. The same is true for the target segment on the right.

As seen in **Figure 4e** to **4h**, at levels where the target volume cannot be separated into disjoint left and right segments, the variant 5-beam arrangement again splits the prescription in 2 halves, that is, using the posterior oblique beams to deliver the first half in uniform, SFO-like dose distributions to the ipsilateral targets, and simultaneously allows MFO dose contributions from the other 3 oblique beams (ie, including the contralateral anterior oblique beams as long as they are not blocked by their respective SAVs owing to metal dental fillings) to deliver the other half of the prescription for better OAR sparing.

### Dosimetric and Robustness Evaluations

The MFOspv/sav plan shows superior OAR sparing over both MFOcon and MFOspv plans. Table 1 shows that while all 3 nominal plans satisfy the physician's requirement on target coverage and OAR dose limits, the MFOcon plan has only 2 OARs with the lowest dose values out of the total 14 OARs (chiasm and left temporal lobe), the MFOspv plan has 3 (cord, left submandibular gland, and right temporal lobe), and the MFOspv/sav has 9. This demonstrated the superiority in OAR sparing for MFOspv/sav.

Robustness evaluations using both simulated scenarios and forward-calculated plans on the weekly VFCT scans confirmed plan robustness for the MFOspv/sav plan. Among all simulated scenarios seen in Table 1 and **Supplemental Figure S1** only 4 of the dose statistics values failed our clinical criteria for the MFOspv/sav, while a total of 17 and 20 dose statistics values failed for the MFOspv and the MFOcon plans, respectively. The averaged overall D1.0cc (Table 1, last column) of the simulated scenarios are 114.5%, 109.6%, and 112.7% of prescription MFOcon, MFOspv, and MFOspv/sav, respectively, and all are within our clinical criteria of 115%. The CTV coverage requirement for the nominal plan is D97% > 99% and the robustness requirement is that the averaged coverage from all 16 scenarios achieve D97% > 94%. All methods meet this minimum CTV coverage requirement.

For forward-calculated VFCT plans, as seen in Table 2 and **Supplemental Figure S2**, target coverages are largely conserved for all MFO plans across all 7 VFCTs, with the averaged CTV56 D97% coverage at 55.9, 55.7, and 55.9 GyRBE, and CTV63 D97% coverage at 62.5, 63.0, and 62.9 GyRBE for MFOspv/sav, MFOspv, and MFOcon, respectively. In addition, dose values for 12 of the 14 OARs are also relatively unchanged from those of their respective nominal plans for all 7 VFCTs. Specifically, the 9 OARs (brainstem, parotids, lacrimal glands, larynx, oral cavity, and cochleae) for which the nominal MFOspv/sav plan has the lowest values all continue to be consistently the lowest among all 3 MFO plans for all VFCTs. The cord maximum dose (Dmax), on the other hand, continues to be the lowest for the MFOspv plan, although all 3 MFO plans consistently achieved Dmax less than 40 GyRBE in all VFCTs. Largest dose variations for all 3 MFO plans are seen in the optic chiasm's D0.05cc values owing to proximity to the target and shape of the OAR. The MFOspv/sav plan still has an advantage for the optic chiasm where its D0.05cc values are less than 54 GyRBE in 2 of the 7 VFCTs, while the MFOcon plan has 1, and the MFOspv plan has all of its chiasm D0.05cc values above 54 GyRBE amongst all VFCTs.

## Discussion

The 5-beam technique typically achieves excellent parotid sparing and provides better sparing for medial OARs such as the oral cavity and pharyngeal constrictor. In addition, the versatility of the 5-beam arrangement to adapt to different target anatomies also broadens its use. As a result, almost all bilateral H&N cases are treated with this 5-beam arrangement in our clinics. Specifically, spot guidance structures and associated planning objectives at levels with (**Figure 2b** to **2d**) and without (**Figure 2a**) medial involvement follow the variant and the standard 5-beam techniques, respectively. The resultant dose distribution at levels with and without medial involvement therefore resembles that of the variant (**Figure 4e** to **4h**) and the standard (**Figure 4a** to **4d**) 5-beam arrangement, respectively. Note that with the 5-beam arrangement, cord maximum dose typically is not the limiting factor and as a result, the SAVs for the posterior obliques are often edited to allow better parotid sparing.

The SFO regions of the AP and PA beams have a CSI-like gradient dose matching in their “transition” region (orange in Figure 1). The “control” region (blue in **Figure 2d**) separating the respective SFO regions of the LPO and RPO in the midline where the target is connected medially acts in the same manner. This CSI-like dose gradient effectively mitigates the potential dose heterogeneity when changes in the day-to-day setup cause beams to “bump into” or “be separated from” each other. Indeed, such dose gradients have successfully mitigated overlaps and/or separations, and reduced hot/cold spots for proton CSI treatments. In addition, the MFOspv/sav technique's integration of SFO regions also specifically limits each individual beam's contribution to any part of the target to half of the prescription. In our experience, when no part of the target is relying on 1 single beam to deliver most of the prescription, the resulting plan is less likely to show large magnitude heterogeneity in forward-calculated VFCT plans.

The 3 MFO planning techniques presented here, that is, MFOcon, MFOspv, and MFOspv/sav, each relies progressively more on user-imposed guidance on top of the optimization process driven by the cost function. The resulting solution spaces for these 3 MFO techniques therefore shrink from MFOcon to MFOspv, and then again from MFOspv to MFOspv/sav. As a result, in theory, with a larger solution space the MFOcon technique indeed does not prevent the TPS from finding the same optimization result as the MFOspv and MFOspv/sav techniques. In practice, however, we have not yet encountered an instance where dose statistics and plan robustness of the MFOspv/sav plan can be achieved by simply using the MFOspv or MFOcon. We surmise that this is because current TPS optimizers do not have a consistent method to reach the desired local minimum without specific user guidance.

Potentially one can optimize with robust Dmax constraints for the OARs in all 3 MFO approaches. However, in our experience, such setting typically results in a much lower OAR Dmax and undercoverage of the CTV in the nominal plan. Iterative adjustment is needed to find the “right” robustness setting. In addition, this iterative trial-and-error process became intractable when managing multiple critical OARs while simultaneously maintaining CTV coverage. The MFOspv/sav method reaches directly the achievable minimum OAR values with the desired CTV coverage without relying on robust Dmax settings.

The robustness observed in all MFO plans in the VFCT evaluations is a result of the setup and range margins added during robust optimization. To include anatomic variations into robust optimization would require a priori model to predict patients' interfractional anatomic changes. Further studies are needed to disentangle the effect of setup errors and range uncertainties from that of anatomic changes.

## Conclusion

The MFOspv/sav technique is a systematic approach using SPV and SAV to guide the optimizer to consistently reach desired OAR dose values and plan robustness. This results in a more efficient planning process with fewer optimizations required to reach the desired dose distribution and less reliance on user experience, which can result in inconsistencies in the resulting plan. As the current TPS still cannot automatically optimize beam angles and spot placements together with spot weights, MFOspv/sav's guidance on spot placements based on anatomy and beam angles leads the optimizer to more consistent plan quality.
