# Supplementary material for: Beam-Specific Spot Guidance and Optimization for PBS Proton Treatment of Bilateral Head and Neck Cancers
Source: Int J Part Ther. 2021 Jun 25;8(1):50–61. doi: 10.14338/IJPT-20-00060.1 (PMC8270101; doi:10.14338/IJPT-20-00060.1)
Supplement: Supplementary file 1 [file ijpt_08-01-30_s01.pdf]

International Journal of Particle Therapy

Beam-specific spot guidance and optimization for PBS proton treatment of bilateral H&N cancers

--Manuscript Draft--

**Manuscript Number:**	THEIJPT-D-20-00060R2
**Article Type:**	Original Article
**Section/Category:**	Particle Therapy for Head and Neck Malignancies
**Keywords:**	Proton Therapy; head and neck cancer; PBS; MFO; Beam-specific spot placement guidance

**Corresponding Author:**	Chang Chang, Ph.D., DABR<br>California Protons Cancer Therapy Center<br>San Diego, CA UNITED STATES
**First Author:**	Karla Leach
**Order of Authors:**	Karla Leach
	Shikui Tang
	Jared Sturgeon
	Andrew K Lee
	Ryan Grover
	Parag Sanghvi
	James Urbanic
	Chang Chang

Abstract:

Purpose: A multi-field optimization (MFO) technique that utilizes beam-specific spot placement volumes (SPV) and spot avoidance volumes (SAV) is introduced for bilateral head and neck (H&N) cancers. These beam specific volumes are used to guide the optimizer to consistently achieve optimal OAR sparing with target coverage and plan robustness.

Methods: Implementation of this technique using a 4-beam, 5-beam, and variant 5-beam arrangement is discussed. The generation of beam-specific SPVs and SAVs derived from target and OARs are shown. The SPVs for select fields are further partitioned into optimization volumes for uniform dose distributions that resemble those of single-field optimization (SFO). A conventional MFO plan that does not use beam-specific spot placement guidance (MFOcon), and an MFO plan that utilizes only beam-specific SPV (MFOspv), are compared with current technique (MFOspv/sav) using both simulated scenarios and forward-calculated plans on weekly VFCT scans.

Results: Dose distribution characteristics of the 4-beam, 5-beam, and variant 5-beam technique are demonstrated with discussion on OAR sparing. When comparing the MFOcon, MFOspv, and MFOspv/sav, the MFOspv/sav is shown to have superior OAR sparing in 9 of the 14 OARs examined. It also shows clinical plan robustness when evaluated using both simulated uncertainty scenarios and forward-calculated weekly verification CTs throughout the 7-week treatment course.

Conclusion: The MFOspv/sav technique is a systematic approach utilizing SPV and SAV to guide the optimizer to consistently reach desired OAR dose values and plan robustness.

Powered by Editorial Manager® and ProduXion Manager® from Aries Systems Corporation

Ref.: Ms. No. THEIJPT-D-20-00060R1
Beam-specific spot guidance and optimization for PBS proton treatment of bilateral H&N cancers
International Journal of Particle Therapy

Dear Dr. Dong,

We thank the reviewers for their useful comments and we have revised the manuscript accordingly. All changes are summarized in this cover letter in blue ink, and specific revisions from the manuscript are cited in *italic* fonts.

Chang Chang, PhD
California Protons / UCSD

Reviewers' comments:

Associate Editor:

The revised manuscript had made many improvements. After reading Reviewer 2's comments, it seems that authors should make additional clarifications for non-Eclipse users. In particular, the limitations of this approach should be discussed. I believe that the approach can improve the planning process; however, it does not imply that this approach has the best robustness (otherwise, a comparative study needs to be done).

We agree with the Associate Editor and have made revisions to better clarify the limitation of this approach for users of different TPSs. Specifically, we have added a paragraph addressing the limitation of this method in the Discussion section and removed any implication that this approach has the best robustness. Specific responses and revisions are listed below in *italic fonts* together with Reviewer comment.

Reviewer #1:
Thank you for revising your manuscript with the edits suggested before. I have no further comments. Thank you for sharing your good work!
We appreciate Reviewer #1's helpful comments and encouraging remarks.

Reviewer #2:

Please see comments below. The revised manuscript is much better than the initial submission, but still could use some work, specifically addressing the methods and the limitations of this work.

We appreciate Reviewer #2's helpful and detailed comments and we have followed the Reviewer's suggestions to revise the manuscript and included a paragraph on the limitations of

this work in the Discussion section in order to specifically address the limitations of this work. Specifically, limitation of this study is added to the fourth and fifth paragraphs of the Discussion section:

Discussion, fourth and fifth paragraph:
"Potentially one can optimize with robust Dmax constraints for the OARs in all three MFO approaches. However, in our experience, such setting typically results in a much lower OAR Dmax and under-coverage of the CTV in the nominal plan. Iterative adjustment is needed to find the "right" robustness setting. In addition, this iterative trial-and-error process became intractable when one needs to manage multiple critical OARs while simultaneously maintaining CTV coverage. The MFOspv/sav method reaches directly the achievable minimum OAR values with the desired CTV coverage without relying on robust Dmax settings."

"The robustness observed in all MFO plans in the VFCT evaluations is a result of the setup and range margins added during robust optimization. To include anatomical variations into robust optimization would require a priori model to predict patients' interfractional anatomical changes. Further studies are needed to disentangle the effect of setup errors and range uncertainties from that of anatomical changes."

Another comment is that the planning methods described are compared using only a single case. This is because the authors are attempting to demonstrate a novel planning method rather then prove it is better for all cases. Consideration if this justifies publication in the IJPT in this form is a challenging question to consider.

This work focused on a new planning approach that can consistently achieve desired target coverage and OAR sparing with clinically acceptable plan robustness. We have used three different bilateral H&N patients, each with different tumor anatomy and etiology, to demonstrate this planning approach. Comparison was also made with other planning approaches that are commonly seen in contemporary proton clinics. The presented method can augment current treatment planning practices before TPSs can specifically optimize beam angles and spot placements simultaneously with spot weights.

General Comments

- When initially defining each of the SFO regions for each techniques, please discuss the relative weightings from each field more clearly. As written, I had to read through it several times to understand this point.

  We have revised the manuscript to better discuss the relative weightings of each field for the SFO regions. Specifically, the following sections are revised,

  Methods, *SPV/SAV planning technique: 4-beam arrangement,* last paragraph:
  "*The dosimetric goal is for the PA beam to provide half of the prescription in the superior neck with the remaining half delivered in MFO distributions by the anterior*

obliques. While in the inferior neck, the AP will deliver half of the prescription and the two anterior obliques provide the other half to their corresponding ipsilateral side of the target."

Methods, *SPV/SAV planning technique: 5-beam arrangement,* first paragraph:
"*… This technique is identical to the 4-beam in the lower neck region in that no posterior beams are used for the inferior neck nodes. Difference exists superiorly where the single PA is replaced by two posterior obliques. As seen in Fig. 2(a), the posterior oblique beam angles are chosen to be parallel to the interface between the ipsilateral targets and parotid. Due to the target separation into distinct left and right sections, the anterior oblique beams SPVs are defined such that no proton spots are placed across midline. This arrangement provides optimal sparing of the various OARs situated along the medial section, such as the oral cavity and pharyngeal constrictor. Without the PA beam, the posterior obliques require individual SFO structures to deliver half the prescribed dose to their respective ipsilateral side targets while the remaining half will be delivered in MFO distributions by ipsilateral anterior oblique and contralateral posterior oblique.*"

Methods, *SPV/SAV planning technique: variant 5-beam arrangement,* first paragraph:
"… An SFO section on the ipsilateral side is defined within each of the posterior oblique beam's SPV, as seen in Fig. 2(b) to (d). The dosimetric goal is again to have the posterior oblique beams deliver half of the prescribed dose uniformly in a SFO-like manner to the ipsilateral side of the target; and the other half of the prescription delivered as MFO from the other three oblique beams to achieve desired OAR sparing."

Results, *The 4-beam arrangement,* first paragraph:
"… This results inthe anterior oblique beams producing SFO-like dose distributions in the inferior neck when their SPVs overlap with the AP beam's SFO region; it also allows MFO dose distributions in the superior neck for better OAR sparing (Fig. 3(c) to (h))."

- In proton planning, we have several things we attempt to account for to ensure CTV coverage. These include set-up errors, range uncertainty, and inter-fractional anatomical inconsistencies. We can address set-up errors and range uncertainty in the planning though robust optimization and robust evaluations, but anatomical inconsistencies we generally cannot, because we don't have a good model on how they will change. When you do your evaluations on the VFCT's, I believe you are actually testing each plan's sensitivity to inconsistent anatomy one needs to keep in mind that one reason the various planning methods remain robust at these different time points is because of the extended margins you added for set-up and range uncertainty. In short, you may be getting inconsistent anatomy robustness as a consequence of other safety margins you added for other reasons during planning. The only way to prove this is to repeat robustness evaluations on the VFCT's and see how those hold up. Doing this more detailed analysis can be considered out of the scope of this work, but I believe this concept should be mentioned in the discussion, or in a "Limits of this Study" paragraph.\

We agree with the Reviewer that since anatomical changes were not specifically included into the robust optimization, the robustness we observed in the VFCT evaluation is a result of the margins added during robust optimization for setup and range uncertainties. We also agree that a separate more detailed study using repeated VFCTs is needed in order to disentangle the effect of setup errors, range uncertainty, and anatomical changes. We have therefore followed the Reviewer's suggestion to revise the manuscript and added a discussion on the limitation of this study as the fifth paragraph in the Discussion section. Specifically,
"The robustness observed in all MFO plans in the VFCT evaluations is a result of the setup and range margins added during robust optimization. To include anatomical variations into robust optimization would require a priori model to predict patients' interfractional anatomical changes. Further studies are needed to disentangle the effect of setup errors and range uncertainties from that of anatomical changes."

You suggest in several statements that the MFOspv/sav is the most robust, but I see several areas where this can be questioned.

We have removed all statements of "most robust". Specifically,
Abstract, *Results:*
"*..., it also shows clinical plan robustness when evaluated using ...*"
Abstract, *Conclusion:*
"*... to consistently reach desired OAR dose values and plan robustness ...*"
Methods, *SPV/SAV planning technique: 4-beam arrangement,* second paragraph*:*
"*... to consistently reach desired OAR dose values and plan robustness ...*"
Results, *Dosimetric and robustness evaluations*, second paragraph:
"Robustness evaluations using both simulated scenarios and forward-calculated plans on the weekly VFCT scans confirmed plan robustness for the MFOspv/sav plan."
Discussion, last paragraph:
"*The MFOspv/sav technique is a systematic approach utilizing SPV and SAV to guide the optimizer to consistently reach desired OAR.*"

- First, your worse case scenarios in Table 1 show the lowest coverage of the CTV D97% in the MFOspv/sav and in Fig S1, the CTV D97% appear to have the largest spread about the mean. Please describe in the text if and/or how you used the robustness evaluation to determine appropriate CTV robustness. If all methods meet your minimum criteria, the differences between methods is not important, but how this was determined is not defined.
  We have revised the manuscript to clarify that in our clinic the CTV coverage requirement for the nominal plan is D97%>99% and the robustness requirement is that the average of all scenario coverage D97%>94%; and all three MFO plans meet the minimum CTV coverage requirement. We have revised the manuscript and included these definitions in the second paragraph of the **Results,** *Dosimetric and robustness evaluations*. Specifically,
  "*... The CTV coverage requirement for the nominal plan is D97%>99% and the robustness requirement is that the averaged coverage from all 16 scenarios achieves D97%>94%. All methods meet this minimum CTV coverage requirement.*"

- Second, you only optimized with robustness on the minimum dose of the CTV. If you used robustness for max dose on other OAR's, the max dose may have been better across the planning methods. For example, Max dose to the chiasm was left as a non-robust objective, so expecting it to be robust is not quite fair. I believe your SPV/SAV technique is artificially (but intentionally) adding robustness that could potentially be accounted for in MFOconv plans if the appropriate objectives were optimized with robustness. The point of this work is that you save this extra work on the optimizer and help solve it using the SPV/SAV methods, which is a noteworthy effort. This is mentioned in the manuscript, but please consider discussing in more detail in the discussion section.

  We have revised the manuscript and added a paragraph to discuss this point in detail in the fourth paragraph of the Discussion. Specifically,
  "Potentially one can optimize with robust Dmax constraints for the OARs in all three MFO approaches. However, in our experience, such setting typically results in a much lower OAR Dmax and under-coverage of the CTV in the nominal plan. Iterative adjustment is needed to find the "right" robustness setting. In addition, this iterative trial-and-error process became intractable when one needs to manage multiple critical OARs while simultaneously maintaining CTV coverage. The MFOspv/sav method reaches directly the achievable minimum OAR values with the desired CTV coverage without relying on robust Dmax settings."

- It appears in your 5 field methods that distal edges from the opposite posterior obliques could be end relatively close to each other. In the case of -4% range uncertainty, this could lead to considerable hot spots and would be something we would worry about in our clinic. It may be worthwhile to add this parameter into Table 1.

  We have revised Tables 1 and 2 to include the D1.0cc for the overall external contour in the last column. The revised table is included in this revision. The averaged hot spots are 114.5%, 109.6%, and 112.7% of Rx for MFOcon, MFOspv and MFOspv/sav, respectively. They are all within our clinical criteria of 115% and the dose homogeneity is largely preserved. We have also revised the manuscript accordingly. Specifically, **Results**, <u>*Dosimetric and robustness evaluations*</u>, second paragraph:
  "... The averaged overall D1.0cc (Table 1, last column) of the simulated scenarios are 114.5%, 109.6%, and 112.7% of Rx MFOcon, MFOspv and MFOspv/sav, respectively, and all are within our clinical criteria of 115%."

- I think that much of the Supplemental Information could be incorporated into the main manuscript. I am not convinced pulling it out as a "supplement" is necessary, but I would defer that decision to the associate editor.

  We have selected the most relevant and important contents of this work to include in the main context as allowed by the manuscript length limit of 4,500 words and 6 displays. We have moved detailed implementation procedures into the Supplement information to help the readers in the case that they implement this planning method in their clinics.

Specific Comments:

- Page 16, Ln 18 : "Proton PBS treatment planning is an optimization process that puts together numerous proton spots of various energies at proper locations" The optimization process also "puts together" the relative weights of the spots. Consider adding this important item.
  We have revised the sentence accordingly. Specifically,
  Introduction, second paragraph:
  "*... that puts together numerous proton spots of various energies at locations with proper weights.*"

- Page 16 Ln 49 : "However, treatment planning systems" consider revising to "However, the treatment planning systems…"
  We have revised the sentence accordingly. Specifically,
  Introduction, third paragraph:
  "*... However, the treatment planning systems (TPS) ...*"

- Page 18 Line 10 : Define the "beam avoidance options" better. This is not available in all planning systems.
  We have revised the manuscript to outline the process when the beam avoidance option is not available. Specifically, in the Method section:
  Methods, *SPV/SAV planning technique: 4-beam arrangement,* fourth paragraph*:*
  "*... For TPS's that do not provide spot avoidance function, a manual process is needed to project the SAV along the beam path to be subtracted from…*"

In addition, we have moved the sections on sinus involvement, i.e. the second paragraph in **Method** *SPV/SAV planning technique: 5-beam arrangement* and the last sentence of the first paragraph in the **Results** *The 5-beam arrangement: standard and variant*, to Supplement due to length limitations. We have also shortened some of the paragraphs to meet the length requirement and added one summarizing sentence to the end of the **Discussion** section. Specifically,
"*As the current TPS still cannot automatically optimize beam angles and spot placements together with spot weights, MFOspv/sav's guidance on spot placements based on anatomy and beam angles leads the optimizer to more consistent plan quality.*"

Manuscript
Click here to access/download;Manuscript;HN_R1_rev9_final.docx

Title: Beam-specific spot guidance and optimization for PBS proton treatment of bilateral H&N cancers

Running Title: Beam-specific spot guidance for PBS H&N

Karla Leach BS[1,2], Shikui Tang PhD[2], Jared Sturgeon MD PhD[2], Andrew K. Lee MD[2], Ryan Grover MD[1,3], Parag Sanghvi MD[1,3], James Urbanic MD[1,3], Chang Chang PhD[1,3]

1. California Protons Cancer Therapy Center, San Diego, CA 92109
2. Texas Center for Proton Therapy, Irving, TX 75063
3. Department of Radiation Medicine and Applied Sciences, University of California, San Diego, CA 92109

Included: Four figures (Figure 1 to 4), two tables (Table 1 and 2), and two Supplemental Figures (Figure S1 and S2).

The authors have no conflicts to disclose.

The authors are willing to share all data presented in this work.

This manuscript did not involve a statistician.

Corresponding author:
Chang Chang, PhD
Associate Professor
Department of Radiation Medicine and Applied Sciences
University of California at San Diego
La Jolla, CA 92109
Tel: 650-861-1080
Fax: 858-578-1144
Email: chang2@health.ucsd.edu

Abstract

Purpose: A multi-field optimization (MFO) technique that utilizes beam-specific spot placement volumes (SPV) and spot avoidance volumes (SAV) is introduced for bilateral head and neck (H&N) cancers. These beam specific volumes are used to guide the optimizer to consistently achieve optimal OAR sparing with target coverage and plan robustness.

Methods: Implementation of this technique using a 4-beam, 5-beam, and variant 5-beam arrangement is discussed. The generation of beam-specific SPVs and SAVs derived from target and OARs are shown. The SPVs for select fields are further partitioned into optimization volumes for uniform dose distributions that resemble those of single-field optimization (SFO). A conventional MFO plan that does not use beam-specific spot placement guidance (MFOcon), and an MFO plan that utilizes only beam-specific SPV (MFOspv), are compared with current technique (MFOspv/sav) using both simulated scenarios and forward-calculated plans on weekly VFCT scans.

Results: Dose distribution characteristics of the 4-beam, 5-beam, and variant 5-beam technique are demonstrated with discussion on OAR sparing. When comparing the MFOcon, MFOspv, and MFOspv/sav, the MFOspv/sav is shown to have superior OAR sparing in 9 of the 14 OARs examined. It also shows clinical plan robustness when evaluated using both simulated uncertainty scenarios and forward-calculated weekly verification CTs throughout the 7-week treatment course.

Conclusion: The MFOspv/sav technique is a systematic approach utilizing SPV and SAV to guide the optimizer to consistently reach desired OAR dose values and plan robustness.

Introduction

Cancers of the head and neck present unique challenges in radiation therapy. A typical head and neck target is surrounded by critical organs-at-risk (OAR) such as the oral cavity, parotids, larynx and spinal cord. Proton therapy has the potential to spare these surrounding OARs by exploiting the characteristics of Bragg peaks, within which most of the radiation energy is deposited and no exit dose beyond (1-4). To best utilize this intrinsic property of proton radiation for patient treatments, appropriate planning techniques must be used (5-8).

Recent advancement in proton delivery techniques has enabled active spot-scanning, referred to as the pencil beam scanning (PBS) modality, to be used routinely in the clinic (9, 10). Proton PBS treatment planning is an optimization process that puts together numerous proton spots of various energies at locations with proper weights. The conversion accuracy from Hounsfield Units (HU) to proton relative stopping power determines the accuracy in spot locations and consequently accuracy in proton dose calculation. This property, referred to as the proton range uncertainty, together with the uncertainties in patient setup, must be taken into account in the planning process. A robust optimization, which takes into account the uncertainties in patient setup and proton ranges, is therefore required for proton PBS treatment planning (11-13).

Different planning techniques have varying effects on not only the dosimetric outcomes but also the resultant plan's robustness against the uncertainties (14, 15). Plans with independently optimized beams (Single-Field Optimization, SFO), where each field contributes a uniform dose over the target, are in general more resilient to errors in patient setup and HU calibration. However, SFO plans are not able to utilize compensating dose distributions from more than one beam to spare OARs. On the other hand, plans that are optimized by simultaneously incorporating contributions from multiple beams (Multi-Field Optimization, MFO) are typically more capable of achieving competing target and OAR dose objectives (16-22). In addition, recent advancements in robust optimization have enabled MFO plans with improved robustness by incorporating setup and range uncertainties into the optimization process (23), and as a result greatly expanded the use of MFO in the clinics.

Proton PBS treatment plans using MFO have shown tremendous potential for H&N cancers (24-29). However, the treatment planning systems (TPS) optimizer relies heavily on user's judgements and inputs which can create inconsistencies in plan quality. In this study, we explore an MFO technique that utilizes beam-specific spot placement volumes (SPV) and spot avoidance volumes (SAV), as well as SFO optimization structures within these spot guidance volumes, to guide the optimizer to find the solution that will consistently achieve optimal OAR sparing while maintaining the desired target coverage and plan robustness. We've also presented three variations of this planning technique using one case in each variation and discussed the circumstances that make these variations most beneficial. Robustness of this planning technique

was evaluated on one clinical case using both simulated scenarios on the original planning CT, and forward-calculated original plans on the patient's subsequent weekly verification CTs (VFCTs) throughout the treatment course.

Methods

All plans are optimized with robust minimum dose objectives set to the CTVs for each prescription level. Identical robustness optimization settings, i.e. 4% range uncertainty and 3mm setup uncertainty, are used for all plans. Inter-beam robust optimization was not used due to prolonged planning time.

SPV/SAV planning technique: 4-beam arrangement

The first case examined is a squamous cell carcinoma of the left soft palate stage T2N2cM0 treated to three dose levels 70/63/56 Gy(RBE) in 35 fractions. A case with this type of volume, which has no separation at midline in the oral cavity region, uses a four field arrangement: anterior-posterior (AP), posterior-anterior (PA), left anterior oblique (LAO) and right anterior oblique (RAO). Gantry angles close to +/-60 degrees and couch rotations of +15/-15 degrees are often associated with the RAO and LAO beams respectively to assist OAR sparing and to avoid the shoulder. A ranger shifter is often needed due to shallow tumor depth and the low energy limitations of the treatment delivery system.

Beam-specific SPV and SAV are used to guide the optimizer in the placement of proton spots to achieve desired OAR sparing and plan robustness. The SPV is derived from the planning target volume (PTV), which in turn is obtained from the clinical target volume (CTV) with an isotropic expansion based on setup uncertainty. For H&N cancers, a margin of 3 mm is typically used. The SPV is used to delimit the largest extent of each beam's spot placement. The exact location of proton spots is determined during optimization by the TPS. Each beam's SAV is derived from the OARs with typically 3 mm margin and adjusted based on the beam angle and proximity to the target. These two volumes, SPV and SAV, are synergistically used to guide beam-specific spot placements.

As seen in Fig. 1(a), the SPV for the AP beam includes only the lower neck region, and its superior border must be at least 1.5 cm below the chin, excluding the oral cavity and avoiding the uncertainty in chin position reproducibility. The SPV for the PA beam is superior of the AP beam's SPV and its inferior border must overlap with the AP beam's SPV (Fig. 1(b)) by at least 2 cm. This 2 cm overlap is slightly larger than the lateral penumbra of proton beams at this shallow depth, and it therefore allows the smooth dose gradients of the AP and PA beams to intersect inside this 'transition' region (orange in Fig. 1). The two anterior oblique beams with minor couch kicks, LAO and RAO, as seen in Fig. 1(c) and (d) are typically used to cover the entire superior-inferior length of the target, but with sections on their respective contralateral side

cropped for better OAR sparing. For example, the RAO beam's SPV ends on the left side where the PTV bifurcates inferior of the left parotid (Fig. 1(c)), and vice versa for the RAO (Fig. 1(d)).

SAVs are used to ensure that proton spots do not traverse, nor stop in front of an OAR. The SAVs used by the LAO and RAO beams restrict spot placement around larynx, parotids, oral cavity, submandibular glands, cochlea, brainstem and the spinal cord. Specifically, the SAVs are generated by expanding the OARs with a 3mm expansion, taking a Boolean union of the expansions, and then subtracting the SPV with a 3mm margin. Depending on the relative anatomy, additional manual editing of the SAV may be required to balance target coverage and OAR sparing. As an example, the SAV for the LAO and RAO beams in a 4-beam plan is shown in Fig. 1(e) and (f). Here both LAO and RAO share the same SAV and it is edited around the oral cavity and submandibular glands to ensure that the medial section of the target is accessible by both beams. In addition, any metal dental fillings will also be included into the SAV so that no proton spots can be placed inside or through the metal. Note that here the spinal cord has an additional 5cm posterior expansion to ensure that the anterior oblique beams do not place spots across the midline from the space posterior of the cord. This arrangement still allows the anterior oblique beams to deliver dose across the midline but only through the space anterior of the cord, i.e. only by traversing inside the target. The SAV for the PA beam overlaps with the SAV of the LAO and RAO shown in Fig. 1(e, f) but does not include the cord portion because the PA beam needs access to the medial target. For TPS's that do not provide spot avoidance function, a manual process is needed to project the SAV along the beam path to be subtracted from the SPV. Additional lateral margins, up to 5 to 8 mm, to the SAV may be required to achieve the same level of OAR sparing due to lateral spot margins.

In addition to the spot placement guidance above, further segmented optimization structures within the SPVs are needed for the optimization of SFO-like dose distributions. Two SFO structures are created for the PA and AP beam at the superior and inferior neck. The dosimetric goal is for the PA beam to provide half of the prescription in the superior neck with the remaining half delivered in MFO distributions by the anterior obliques. While in the inferior neck, the AP will deliver half of the prescription and the two anterior obliques provide the other half to their corresponding ipsilateral side of the target. Note that the transition region between the AP-PA beams is specifically left out of these SFO structures to allow the AP and PA beams to fade toward the superior and inferior directions, respectively. This dose distribution emulates that of a craniospinal irradiation (CSI) and provides a smooth dose gradient into the transition region, thus alleviating the potential dose heterogeneity due to various uncertainties (30).

SPV/SAV planning technique: 5-beam arrangement

The second case examined is a sinonasal undifferentiated carcinoma T2N0M0 treated with two dose levels 63/56 Gy(RBE) in 35 fractions. A case with this type of volume where the target is separated at midline around the oral cavity is best suited for the 5-beam technique. This

technique is identical to the 4-beam in the lower neck region in that no posterior beams are used for the inferior neck nodes. Difference exists superiorly where the single PA is replaced by two posterior obliques. As seen in Fig. 2(a), the posterior oblique beam angles are chosen to be parallel to the interface between the ipsilateral targets and parotid. Due to the target separation into distinct left and right sections, the anterior oblique beams' SPVs are defined such that no proton spots are placed across midline. This arrangement provides optimal sparing of the various OARs situated along the medial section, such as the oral cavity and pharyngeal constrictor. Without the PA beam, the posterior obliques require individual SFO structures to deliver half the prescribed dose to their respective ipsilateral side targets while the remaining half will be delivered in MFO distributions by ipsilateral anterior oblique and contralateral posterior oblique.

SPV/SAV planning technique: variant 5-beam arrangement

The third case examined is a squamous cell carcinoma of the right base of tongue stage T4N2bM0 stage III treated to three dose levels 70/63/56 Gy(RBE) in 35 fractions. This treatment volume is similar to the first case (treated with the 4-field arrangement) in that it is connected at midline in the oral cavity region. However, portions of the target are surrounded by metal dental fillings which prevent access by the anterior beams. The 4-beam arrangement is therefore not applicable since both posterior obliques are needed to capture targets posterior of the metal dental fillings and we do not use a single beam to deliver full prescription to any part of the target. A variant 5-beam technique is therefore used where all four oblique beams can place spots across the midline unless otherwise blocked by their respective SAVs. An SFO section on the ipsilateral side is defined within each of the posterior oblique beam's SPV, as seen in Fig. 2(b) to (d). The dosimetric goal is again to have the posterior oblique beams deliver half of the prescribed dose uniformly in a SFO-like manner to the ipsilateral side of the target; and the other half of the prescription delivered as MFO from the other three oblique beams to achieve desired OAR sparing. Also seen in Fig. 2(d) is a 2cm wide 'control' region (blue) separating the two SFO sections (green and red) of the posterior oblique beams. Like the transition region (orange) in Fig. 1(a) to (d), this control region permits proper dose gradients for the two uniform dose distributions from the SFO sections and avoids dose heterogeneity at the junction due to setup, range, and anatomical uncertainties.

Dosimetric and Robustness evaluations

For comparison, the second bilateral head and neck case with sinus involvement was re-planned using two additional MFO techniques: conventional MFO (MFOcon) without any SPV or SAV volumes and MFO with only SPV volumes (MFOspv). These additional MFO techniques are commonly used in PBS treatment planning for head and neck cancers. All plans used the same five beams and robustness settings. The optimization objectives on the original target and OAR contours are identical between the MFOcon, MFOspv, and MFOspv/sav plans. Detailed planning objectives are included in Supplemental Table S1. All three MFO plans are normalized such that

97% of the CTV63 volume is covered by prescription, i.e. D97% = 63 Gy(RBE). These three plans are then designated as the nominal plans in Tables 1 and 2.

Plan robustness for these three MFO plans were evaluated using both simulated uncertainty scenarios and forward-calculated plans on VFCTs taken over the course of the patient's treatment. The robustness evaluation shown in Table 1 and Fig. S1 is part of the standard physics check for all patients before treatment starts. For each uncertainty scenario shown in Table 1, robustness of these three MFO plans was evaluated by deliberately shifting the location of the isocenter by +/-0.3 cm in the x, y and z directions to simulate setup errors, together with +/-4% density perturbation to account for range uncertainties. For example, Scenario 1 corresponds to an isocenter shift of 3mm to the right, 3mm to the anterior, and 3mm to the inferior, as well as a 4% over-range in HU-to-stopping power calibration. A total of 16 different scenarios are evaluated.

Weekly VFCTs were taken during the treatment course. These VFCTs were registered to the planning CT, and the various target and OAR structures were transferred to the VFCTs and reviewed by the attending physician. The three nominal MFO plans were then forward-calculated on these VFCTs for inter-fractional robustness evaluation. The results are summarized in Table 2 and Fig. S2. Values that do not meet our clinical criteria are highlighted in yellow.

Results

The 4-beam arrangement

Dose distributions for the 4-beam technique are shown in Fig. 3. As seen in Fig. 3(a, b), the uniform dose objectives ensured that the AP and PA beams delivered a uniform, i.e. SFO-like, dose distribution to their respective SFO structures within each SPV (excluding the transition region). Note that the PA beam's dose distribution shows three target dose levels (CTV56, CTV63, and CTV70) since this part of the target has simultaneous integrated boost. The AP beam on the other hand treats the inferior nodes and has only one target dose level (CTV56). Note that SFO structures must be created separately for each dose level. A uniform dose objective set to the overall PTV helps ensure overall dose homogeneity and guides the LAO and RAO beams to deliver the remaining half of the prescription using the uniform dose distributions from AP and PA as a baseline. This results in the anterior oblique beams producing SFO-like dose distributions in the inferior neck when their SPVs overlap with the AP beam's SFO region; it also allows MFO dose distributions in the superior neck for better OAR sparing (Fig. 3(c) to (h)). This beam arrangement prevents dose spillage across the midline in the inferior neck and avoids entering the parotids in the superior neck for maximum OAR sparing.

The 5-beam arrangements: standard and variant

Dose distributions for the 5-beam techniques are shown in Fig. 4. Unlike the 4-beam technique, the posterior oblique beams in the 5-beam arrangement are also used to treat around the spinal cord to the contralateral side behind the contralateral parotids. The posterior oblique beams' SAVs don't include a posterior extension of the spinal cord (and brainstem if applicable). As demonstrated in Fig. 4(a) to (d) using standard 5-beam technique, at levels where the target volume can be separated into disjoint left and right segments, the segment on the left receives half of the prescription uniformly (i.e. SFO-like) from the LPO beam, and the other half of the prescribed dose is contributed by an MFO combination from the RPO and LAO beams. The contralateral anterior oblique beam, i.e. RAO, does not contribute to the target on the left in this case. The same is true for the target segment on the right.

As seen in Fig. 4(e) to (h), at levels where the target volume cannot be separated into disjoint left and right segments, the variant 5-beam arrangement again splits the prescription in two halves, i.e. using the posterior oblique beams to deliver the first half in uniform, SFO-like dose distributions to the ipsilateral targets, and simultaneously allows MFO dose contributions from the other three oblique beams (i.e. including the contralateral anterior oblique beams as long as they are not blocked by their respective SAVs due to metal dental fillings) to deliver the other half of the prescription for better OAR sparing.

<u>Dosimetric and robustness evaluations</u>

The MFOspv/sav plan shows superior OAR sparing over both MFOcon and MFOspv plans. Table 1 shows that while all three nominal plans satisfy the physician's requirement on target coverage and OAR dose limits, the MFOcon plan has only two OARs with the lowest dose values out of the total 14 OARs (chiasm and left temporal lobe), the MFOspv plan has three (cord, left submandibular gland, and right temporal lobe), and the MFOspv/sav has nine. This demonstrated the superiority in OAR sparing for MFOspv/sav.

Robustness evaluations using both simulated scenarios and forward-calculated plans on the weekly VFCT scans confirmed plan robustness for the MFOspv/sav plan. Among all simulated scenarios seen in Table1 and Fig. S1 only four of the dose statistics values failed our clinical criteria for the MFOspv/sav, while a total of 17 and 20 dose statistics values failed for the MFOspv and the MFOcon plans, respectively. The averaged overall D1.0cc (Table 1, last column) of the simulated scenarios are 114.5%, 109.6%, and 112.7% of Rx MFOcon, MFOspv and MFOspv/sav, respectively, and all are within our clinical criteria of 115%. The CTV coverage requirement for the nominal plan is D97%>99% and the robustness requirement is that the averaged coverage from all 16 scenarios achieves D97%>94%. All methods meet this minimum CTV coverage requirement.

For forward-calculated VFCT plans, as seen in Table2 and Fig. S2, target coverages are largely conserved for all MFO plans across all 7 VFCTs, with the averaged CTV56 D97%

coverage at 55.9, 55.7, and 55.9 Gy(RBE), and CTV63 D97% coverage at 62.5, 63.0, and 62.9 Gy(RBE) for MFOspv/sav, MFOspv, and MFOcon, respectively. In addition, dose values for 12 of the 14 OARs are also relatively unchanged from those of their respective nominal plans for all 7 VFCTs. Specifically, the 9 OARs (brainstem, parotids, lacrimal glands, larynx, oral cavity, and cochleae) for which the nominal MFOspv/sav plan has the lowest values, all continue to be consistently the lowest among all three MFO plans for all VFCTs. The cord maximum dose (Dmax), on the other hand, continues to be the lowest for the MFOspv plan, although all three MFO plans consistently achieved Dmax less than 40 Gy(RBE) in all VFCTs. Largest dose variations for all three MFO plans are seen in the optic chiasm's D0.05cc values due to proximity to the target and shape of the OAR. The MFOspv/sav plan still has an advantage for the optic chiasm where its D0.05cc vales are less than 54 Gy(RBE) in two of the seven VFCTs, while the MFOcon plan has one and the MFOspv plan has all of its chiasm D0.05cc values over 54 Gy(RBE) amongst all VFCTs.

Discussions

The 5-beam technique typically achieves excellent parotid sparing and provides better sparing for medial OARs such as the oral cavity and pharyngeal constrictor. In addition, the versatility of the 5-beam arrangement to adapt to different target anatomies also broadens its use. As a result, almost all bilateral H&N cases are treated with this 5-beam arrangement in our clinics. Specifically, spot guidance structures and associated planning objectives at levels with (Fig. 2(b) to (d)) and without (Fig. 2(a)) medial involvement follow the variant and the standard 5-beam techniques, respectively. The resultant dose distribution at levels with and without medial involvement therefore resembles that of the variant (Fig. 4(e) to (h)) and the standard (Fig. 4 (a) to (d)) 5-beam arrangement, respectively. Note that with the 5-beam arrangement, cord maximum dose typically is not the limiting factor and as a result, the SAVs for the posterior obliques are often edited to allow better parotid sparing.

The SFO regions of the AP and PA beams have a CSI-like gradient dose matching in their 'transition' region (orange in Fig. 1). The 'control' region (blue in Fig. 2(d)) separating the respective SFO-regions of the LPO and RPO in the midline where the target is connected medially act in the same manner. This CSI-like dose gradient effectively mitigates the potential dose heterogeneity when changes in the day-to-day setup cause beams to "bump into" or "be separated from" each other. Indeed, such dose gradients have successfully mitigated overlaps and/or separations, and reduced hot/cold spots for proton CSI treatments. In addition, the MFOspv/sav technique's integration of SFO regions also specifically limits each individual beam's contribution to any part of the target to half of the prescription. In our experience, when no part of the target is relying on one single beam to deliver the majority of the prescription, the resulting plan is less likely to show large magnitude heterogeneity in forward-calculated VFCT plans.

The three MFO planning techniques presented here, i.e. MFOcon, MFOspv, and MFOspv/sav, each relies progressively more on user-imposed guidance on top of the optimization process driven by the cost function. The resulting solution spaces for these three MFO techniques therefore shrink from MFOcon to MFOspv, and then again from MFOspv to MFOspv/sav. As a result, in theory, with a larger solution space the MFOcon technique indeed does not prevent the TPS from finding the same optimization result as the MFOspv and MFOspv/sav techniques. In practice, however, we have not yet encountered an instance where dose statistics and plan robustness of the MFOspv/sav plan can be achieved by simply using the MFOspv or MFOcon. We surmise that this is because current TPS optimizers do not have a consistent method to reach the desired local minimum without specific user guidance.

Potentially one can optimize with robust Dmax constraints for the OARs in all three MFO approaches. However, in our experience, such setting typically results in a much lower OAR Dmax and under-coverage of the CTV in the nominal plan. Iterative adjustment is needed to find the "right" robustness setting. In addition, this iterative trial-and-error process became intractable when one needs to manage multiple critical OARs while simultaneously maintaining CTV coverage. The MFOspv/sav method reaches directly the achievable minimum OAR values with the desired CTV coverage without relying on robust Dmax settings.

The robustness observed in all MFO plans in the VFCT evaluations is a result of the setup and range margins added during robust optimization. To include anatomical variations into robust optimization would require *a priori* model to predict patients' interfractional anatomical changes. Further studies are needed to disentangle the effect of setup errors and range uncertainties from that of anatomical changes.

The MFOspv/sav technique is a systematic approach utilizing SPV and SAV to guide the optimizer to consistently reach desired OAR dose values and plan robustness. This results in a more efficient planning process with fewer optimizations required to reach the desired dose distribution and less reliance on user experience which can result in inconsistencies in the resulting plan. As the current TPS still cannot automatically optimize beam angles and spot placements together with spot weights, MFOspv/sav's guidance on spot placements based on anatomy and beam angles leads the optimizer to more consistent plan quality.

Figure 1: View of (a) AP, (b) PA, (c) RAO and (d) LAO spot placement volumes (yellow, blue, green, and pink, respectively) with the transition region in orange. The SPV for LAO is in pink shade and RAO in green outline (e, f). Both RAO and LAO share the same SAV (in blue outline). The SPVs for both RAO and LAO are connected (e) across the midline and separated (f) below the parotids.

Figure 2: (a) Beam arrangement for the 5-beam techniques. SAVs are shown in green. (b) SPV (pink) and SAV (green) for the LPO. (c) SPV (pink) and SAV (orange) for the RPO beam. (d) SFO sections of RPO and LPO are separated by a 2 cm gap at midline. The SFO section of the RPO (green) and the SFO section of the LPO (red) are seen to be separated by a 2 cm control region (blue).

Figure 3: Dose distributions of the AP (a) and PA (b) beams (coronal), and the RAO (c, f), PA (b, d) and LAO (e, h) beams (axial) for the 4-beam technique. For the superior portion of the target (c, d, e), half of the prescription is delivered through the PA beam using a uniform dose criterion. Only AP, RAO and LAO are used for the inferior portion of the target (f, g, h).

Figure 4: Dose distributions of (a) RPO, (b) RAO, (c) LAO and (d) LPO beams for the 5-beam technique at levels where the target can be separated into left and right segments. Dose distributions from the (e) RPO, (f) RAO, (g) LPO and (h) LAO beams for the variant 5-beam technique at levels where the target has medial involvement.

Table 1: Robustness evaluation of the three MFO planning techniques using simulated scenarios.

Table 2: Inter-fraction plan robustness evaluation of the three MFO planning techniques using VFCTs taken weekly over the treatment course.

1. P. Blanchard *et al.*, Proton Therapy for Head and Neck Cancers. *Semin Radiat Oncol* **28**, 53-63 (2018).
2. A. C. Moreno *et al.*, Intensity modulated proton therapy (IMPT) - The future of IMRT for head and neck cancer. *Oral Oncol* **88**, 66-74 (2019).
3. X. Li, A. Lee, M. A. Cohen, E. J. Sherman, N. Y. Lee, Past, present and future of proton therapy for head and neck cancer. *Oral Oncol* **110**, 104879 (2020).
4. P. DeLuca Jr, A. Wambersie, G. Whitmore, ICRU Report 78: Prescribing, Recording, and Reporting Proton-Beam Therapy. *J ICRU* **7**, 1-210 (2007).
5. H. Paganetti, Range uncertainties in proton therapy and the role of Monte Carlo simulations. *Phys Med Biol* **57**, R99-117 (2012).
6. J. Unkelbach *et al.*, Robust radiotherapy planning. *Phys Med Biol* **63**, 22TR02 (2018).
7. A. J. Lomax, Myths and realities of range uncertainty. *Br J Radiol* **93**, 20190582 (2020).
8. K. Langen, M. Zhu, Concepts of PTV and Robustness in Passively Scattered and Pencil Beam Scanning Proton Therapy. *Semin Radiat Oncol* **28**, 248-255 (2018).
9. R. Mohan, I. J. Das, C. C. Ling, Empowering Intensity Modulated Proton Therapy Through Physics and Technology: An Overview. *Int J Radiat Oncol Biol Phys* **99**, 304-316 (2017).
10. A. J. Lomax *et al.*, Intensity modulated proton therapy: a clinical example. *Med Phys* **28**, 317-324 (2001).
11. W. Liu, X. Zhang, Y. Li, R. Mohan, Robust optimization of intensity modulated proton therapy. *Med Phys* **39**, 1079-1091 (2012).
12. W. Gu *et al.*, Robust optimization for intensity-modulated proton therapy with soft spot sensitivity regularization. *Med Phys* **46**, 1408-1425 (2019).
13. J. Ma *et al.*, Robust radiobiological optimization of ion beam therapy utilizing Monte Carlo and microdosimetric kinetic model. *Phys Med Biol* **65**, 155020 (2020).
14. S. J. Frank *et al.*, Multifield optimization intensity modulated proton therapy for head and neck tumors: a translation to practice. *Int J Radiat Oncol Biol Phys* **89**, 846-853 (2014).
15. A. Anand, M. Bues, M. E. Gamez, C. Stefan, S. H. Patel, Individual Field Simultaneous Optimization (IFSO) in spot scanning proton therapy of head and neck cancers. *Med Dosim* **44**, 375-378 (2019).
16. K. H. Kang *et al.*, Proton therapy for head and neck paragangliomas: A single institutional experience. *Head Neck* **42**, 670-677 (2020).
17. A. Bolsi *et al.*, Pencil beam scanning proton therapy for the treatment of craniopharyngioma complicated with radiation-induced cerebral vasculopathies: A dosimetric and linear energy transfer (LET) evaluation. *Radiother Oncol* **149**, 197-204 (2020).
18. M. J. Pelak *et al.*, Clinical outcomes of head and neck adenoid cystic carcinoma patients treated with pencil beam-scanning proton therapy. *Oral Oncol* **107**, 104752 (2020).
19. M. Yoon *et al.*, Craniospinal irradiation techniques: a dosimetric comparison of proton beams with standard and advanced photon radiotherapy. *Int J Radiat Oncol Biol Phys* **81**, 637-646 (2011).
20. U. Mock, D. Georg, J. Bogner, T. Auberger, R. Pötter, Treatment planning comparison of conventional, 3D conformal, and intensity-modulated photon (IMRT) and proton therapy for paranasal sinus carcinoma. *Int J Radiat Oncol Biol Phys* **58**, 147-154 (2004).
21. A. J. Lomax, E. Pedroni, H. Rutz, G. Goitein, The clinical potential of intensity modulated proton therapy. *Z Med Phys* **14**, 147-152 (2004).
22. L. Cozzi, A. Fogliata, A. Lomax, A. Bolsi, A treatment planning comparison of 3D conformal therapy, intensity modulated photon therapy and proton therapy for treatment of advanced head and neck tumours. *Radiother Oncol* **61**, 287-297 (2001).

23. K. Stützer, A. Lin, M. Kirk, L. Lin, Superiority in Robustness of Multifield Optimization Over Single-Field Optimization for Pencil-Beam Proton Therapy for Oropharynx Carcinoma: An Enhanced Robustness Analysis. *Int J Radiat Oncol Biol Phys* **99**, 738-749 (2017).
24. A. Beddok *et al.*, Proton therapy for head and neck squamous cell carcinomas: A review of the physical and clinical challenges. *Radiother Oncol* **147**, 30-39 (2020).
25. A. S. Grewal, J. Jones, A. Lin, Palliative Radiation Therapy for Head and Neck Cancers. *Int J Radiat Oncol Biol Phys* **105**, 254-266 (2019).
26. A. Lee *et al.*, Trends and Disparities of Proton Therapy Use among Patients with Head and Neck Cancer: Analysis from the National Cancer Database (2005-14). *Int J Part Ther* **5**, 1-10 (2019).
27. J. E. Leeman *et al.*, Proton therapy for head and neck cancer: expanding the therapeutic window. *Lancet Oncol* **18**, e254-e265 (2017).
28. G. B. Gunn *et al.*, Clinical Outcomes and Patterns of Disease Recurrence After Intensity Modulated Proton Therapy for Oropharyngeal Squamous Carcinoma. *Int J Radiat Oncol Biol Phys* **95**, 360-367 (2016).
29. A. J. Lomax, M. Goitein, J. Adams, Intensity modulation in radiotherapy: photons versus protons in the paranasal sinus. *Radiother Oncol* **66**, 11-18 (2003).
30. H. Lin *et al.*, Supine craniospinal irradiation using a proton pencil beam scanning technique without match line changes for field junctions. *Int J Radiat Oncol Biol Phys* **90**, 71-78 (2014).

Title: Beam-specific spot guidance and optimization for PBS proton treatment of bilateral H&N cancers

Running Title: Beam-specific spot guidance for PBS H&N

Karla Leach BS[1,2], Shikui Tang PhD[2], Jared Sturgeon MD PhD[2], Andrew K. Lee MD[2], Ryan Grover MD[1,3], Parag Sanghvi MD[1,3], James Urbanic MD[1,3], Chang Chang PhD[1,3]

1. California Protons Cancer Therapy Center, San Diego, CA 92109
2. Texas Center for Proton Therapy, Irving, TX 75063
3. Department of Radiation Medicine and Applied Sciences, University of California, San Diego, CA 92109

Included: Four figures (Figure 1 to 4), two tables (Table 1 and 2), and two Supplemental Figures (Figure S1 and S2).

The authors have no conflicts to disclose.

The authors are willing to share all data presented in this work.

This manuscript did not involve a statistician.

Corresponding author:
Chang Chang, PhD
Associate Professor
Department of Radiation Medicine and Applied Sciences
University of California at San Diego
La Jolla, CA 92109
Tel: 650-861-1080
Fax: 858-578-1144
Email: chang2@health.ucsd.edu

Abstract

Purpose: A multi-field optimization (MFO) technique that utilizes beam-specific spot placement volumes (SPV) and spot avoidance volumes (SAV) is introduced for bilateral head and neck (H&N) cancers. These beam specific volumes are used to guide the optimizer to consistently achieve optimal OAR sparing with target coverage and plan robustness.

Methods: Implementation of this technique using a 4-beam, 5-beam, and variant 5-beam arrangement is discussed. The generation of beam-specific SPVs and SAVs derived from target and OARs are shown. The SPVs for select fields are further partitioned into optimization volumes for uniform dose distributions that resemble those of single-field optimization (SFO). A conventional MFO plan that does not use beam-specific spot placement guidance (MFOcon), and an MFO plan that utilizes only beam-specific SPV (MFOspv), are compared with current technique (MFOspv/sav) using both simulated scenarios and forward-calculated plans on weekly VFCT scans.

Results: Dose distribution characteristics of the 4-beam, 5-beam, and variant 5-beam technique are demonstrated with discussion on OAR sparing. When comparing the MFOcon, MFOspv, and MFOspv/sav, the MFOspv/sav is shown to have superior OAR sparing in 9 of the 14 OARs examined. ~~More importantly, i~~It also shows ~~superior~~clinical plan robustness when evaluated using both simulated uncertainty scenarios and forward-calculated weekly verification CTs throughout the 7-week treatment course.

Conclusion: The MFOspv/sav technique is a systematic approach utilizing SPV and SAV to guide the optimizer to consistently reach ~~optimal~~desired OAR dose values and plan robustness.

Introduction

Cancers of the head and neck present unique challenges in radiation therapy. A typical head and neck target is surrounded by critical organs-at-risk (OAR) such as the oral cavity, parotids, larynx and spinal cord. Proton therapy has the potential to spare these surrounding OARs by exploiting the characteristics of Bragg peaks, within which most of the radiation energy is deposited and no exit dose beyond (1-4). To best utilize this intrinsic property of proton radiation for patient treatments, appropriate planning techniques must be used (5-8).

Recent advancement in proton delivery techniques has enabled active spot-scanning, ~~sometimes~~ referred to as the pencil beam scanning (PBS) modality, to be used routinely in the clinic (9, 10). Proton PBS treatment planning is an optimization process that puts together numerous proton spots of various energies at ~~proper~~ locations with proper weights. The conversion accuracy from Hounsfield Units (HU) to proton relative stopping power determines the accuracy in spot locations and consequently accuracy in proton dose calculation. This property, ~~often~~ referred to as the proton range uncertainty, together with the uncertainties in patient setup, must be taken into account in the planning process. A robust optimization, which takes into account the uncertainties in patient setup and proton ranges ~~concurrently during optimization~~, is therefore required for proton PBS treatment planning (11-13).

Different planning techniques have varying effects on not only the dosimetric outcomes but also the resultant plan's robustness against the uncertainties (14, 15). Plans with independently optimized beams (Single-Field Optimization, SFO), where each field contributes a uniform dose over the target, are in general more resilient to errors in patient setup and HU calibration. However, SFO plans are not able to utilize compensating dose distributions from more than one beam to spare OARs. On the other hand, plans that are optimized by simultaneously incorporating contributions from multiple beams (Multi-Field Optimization, MFO) are typically more capable of achieving competing target and OAR dose objectives (16-22). In addition, recent advancements in robust optimization have enabled MFO plans with improved robustness by incorporating setup and range uncertainties into the optimization process (23), and as a result greatly expanded the use of MFO in the clinics.

Proton PBS treatment plans using MFO have shown tremendous potential for H&N cancers (24-29). However, the treatment planning systems (TPS) optimizer relies heavily on user's judgements and inputs which can create inconsistencies in plan quality. In this study, we explore an MFO technique that utilizes beam-specific spot placement volumes (SPV) and spot avoidance volumes (SAV), as well as SFO optimization structures within these spot guidance volumes, ~~in order~~ to guide the optimizer to find the solution that will consistently achieve optimal OAR sparing while maintaining the desired target coverage and plan robustness. We've also presented three variations of this planning technique using one case in each variation and discussed the circumstances that make these variations most beneficial. Robustness of this

planning technique was evaluated on one clinical case using both simulated scenarios on the original planning CT, and forward-calculated original plans on the patient's subsequent weekly verification CTs (VFCTs) throughout the treatment course.

Methods

All plans are optimized with robust minimum dose objectives set to the CTVs for each prescription level. Identical robustness optimization settings, i.e. 4% range uncertainty and 3mm setup uncertainty, are used for all plans. Inter-beam robust optimization was not used due to prolonged planning time.

SPV/SAV planning technique: 4-beam arrangement

The first case examined is a squamous cell carcinoma of the left soft palate stage T2N2cM0 treated to three dose levels 70/63/56 Gy(RBE) in 35 fractions. A case with this type of volume, which has no separation at midline in the oral cavity region, uses a ~~typical~~ four ~~beam~~ field arrangement~~ of four fields~~: anterior-posterior (AP), posterior-anterior (PA), left anterior oblique (LAO) and right anterior oblique (RAO). Gantry angles close to +/-60 degrees and couch rotations of +15/-15 degrees are often associated with the RAO and LAO beams respectively to assist OAR sparing and to avoid the shoulder. A ranger shifter is often needed due to shallow tumor depth and the low energy limitations of the treatment delivery system.

Beam-specific SPV and SAV are used to guide the optimizer in the placement of proton spots to achieve ~~optimal~~desired OAR sparing and plan robustness. The SPV is derived from the planning target volume (PTV), which in turn is obtained from the clinical target volume (CTV) with an isotropic expansion based on setup uncertainty. For H&N cancers, a margin of 3 mm is typically used. The SPV is used to delimit the largest extent of each beam's spot placement. The exact location of proton spots is determined during optimization by the TPS. Each beam's SAV is derived from the OARs with typically 3 mm margin and adjusted based on the beam angle and proximity to the target. These two volumes, SPV and SAV, are synergistically used to guide beam-specific spot placements.

As seen in Fig. 1(a), the SPV for the AP beam includes only the lower neck region, and its superior border must be at least 1.5 cm below the chin, excluding the oral cavity and avoiding the ~~associated~~ uncertainty in chin position reproducibility. The SPV for the PA beam is superior of the AP beam's SPV and its inferior border must overlap with the AP beam's SPV (Fig. 1(b))~~. For most bilateral H&N cases, we use~~ by at least 2 cm ~~of overlap between the two SPV's~~. This 2 cm overlap is slightly larger than the lateral penumbra of proton beams at this shallow depth, and it therefore allows the smooth dose gradients of the AP and PA beams to intersect inside this 'transition' region (orange in Fig. 1). The two anterior oblique beams with minor couch kicks, LAO and RAO, as seen in Fig. 1(c) and (d) are typically used to cover the entire superior-inferior

length of the target, but with sections on their respective contralateral side cropped for better OAR sparing. For example, the RAO beam's SPV ends on the left side where the PTV bifurcates inferior of the left parotid (Fig. 1(c)), and vice versa for the RAO (Fig. 1(d)).

SAVs are used to ensure that proton spots do not traverse, nor stop in front of an OAR. The SAVs used by the LAO and RAO beams restrict spot placement around larynx, parotids, oral cavity, submandibular glands, cochlea, brainstem and the spinal cord. Specifically, the SAVs are generated by expanding the OARs with a 3mm expansion, taking a Boolean union of the expansions, and then subtracting the SPV with a 3mm margin. Depending on the relative anatomy, additional manual editing of the SAV may be required to balance target coverage and OAR sparing. As an example, the SAV for the LAO and RAO beams in a 4-beam plan is shown in Fig. 1(e) and (f). Here both LAO and RAO share the same SAV and it is edited around the oral cavity and submandibular glands to ensure that the medial section of the target is accessible by both beams. In addition, any metal dental fillings will also be included into the SAV so that no proton spots can be placed inside or through the metal. Note that here the spinal cord has an additional 5cm posterior expansion to ensure that the anterior oblique beams do not place spots across the midline from the space posterior of the cord. This arrangement still allows the anterior oblique beams to deliver dose across the midline but only through the space anterior of the cord, i.e. only by traversing inside the target. The SAV for the PA beam overlaps with the SAV of the LAO and RAO shown in Fig. 1(e, f) but does not include the cord portion because the PA beam needs access to the medial target. For TPS's that do not provide spot avoidance function, a manual process is needed to~~one can~~ project the SAV along the beam path to be subtracted from the SPV. Additional lateral margins, up to 5 to 8 mm, to the SAV may be required to achieve the same level of OAR sparing due to lateral spot margins.

In addition to the spot placement guidance above, further segmented optimization structures within the SPVs are ~~also~~ needed ~~to provide planning objectives~~ for the optimization of SFO-like dose distributions. Two SFO structures are created for the PA and AP beam at the superior and inferior neck. The dosimetric goal is ~~F~~for the ~~superior neck, the~~ PA beam to provide~~s~~ half of the prescription ~~uniformly~~in the superior neck ~~and~~with the remaining half delivered ~~as~~in MFO distributions- by the anterior obliques. While in ~~For~~ the inferior neck, the AP will deliver~~s~~ half of the prescription ~~uniformly~~ and the two anterior obliques provide the other half ~~uniformly~~ to their corresponding ipsilateral side of the target. Note that the transition region between the AP-PA beams is specifically left out of these SFO structures to allow the AP and PA beams to fade toward the superior and inferior directions, respectively. This dose distribution emulates that of a craniospinal irradiation (CSI) and provides a smooth dose gradient into the transition region, thus alleviating the potential dose heterogeneity due to various uncertainties (30).

SPV/SAV planning technique: 5-beam arrangement

The second case examined is a sinonasal undifferentiated carcinoma T2N0M0 treated with two dose levels 63/56 Gy(RBE) in 35 fractions. A case with this type of volume where the target is separated at midline around the oral cavity is best suited for the 5-beam technique. This ~~e 5-beam~~ technique is identical to ~~its~~the 4-beam ~~counterpart~~ in the lower neck region ~~since~~in that no posterior beams are used for the inferior neck nodes. Difference exists superiorly where the single PA is replaced by two posterior obliques. ~~It is a variation of the 4-beam technique where the PA beam is replaced by two posterior oblique beams. This arrangement provides optimal sparing of the various OARs situated along the medial section, such as the oral cavity and pharyngeal constrictor.~~ As seen in Fig. 2(a), the posterior oblique beam angles are chosen to be parallel to the interface between the ipsilateral targets and parotid. ~~The 5-beam technique is identical to its 4-beam counterpart in the lower neck region since no posterior beams are used for the inferior neck nodes. Difference exists superiorly where the single PA is replaced by two posterior obliques.~~ Due to the target separation into distinct left and right sections, the anterior oblique beams SPVs are defined such that no proton spots are ~~not allowed to~~ placed ~~spots~~ across ~~the~~ midline. This arrangement provides optimal sparing of the various OARs situated along the medial section, such as the oral cavity and pharyngeal constrictor. Without the PA beam, the posterior obliques require individual SFO structures ~~are used~~ to deliver half the prescribed dose to their respective ipsilateral side targets while the remaining half will be delivered in MFO distributions by ipsilateral anterior oblique and contralateral posterior oblique.

~~For the sinus region, an SFO structure is created for the AP beam excluding regions of the PTV that are posterior to the eyes. The SAV in the sinus region includes the eyes, brainstem, and cochlea. The AP beam is typically tilted by about +/- 3 degrees to prevent the beam from being tangent to the bone and air interface in the sinus. For cases where the target volume extends to spaces behind the eyes, the eye and temporal lobe doses can become a concern, and a sixth non-coplanar beam from the superior anterior direction can be used to reduce the eye and temporal lobe dose.~~

SPV/SAV planning technique: variant 5-beam arrangement

The third case examined is a squamous cell carcinoma of the right base of tongue stage T4N2bM0 stage III treated to three dose levels 70/63/56 Gy(RBE) in 35 fractions. This treatment volume is ~~otherwise~~ similar to the first case ~~above~~ (treated with the 4-field arrangement) in that it is connected at midline in the oral cavity region. However, portions of the target are surrounded by metal dental fillings which prevent access by the anterior beams. The 4-beam arrangement is therefore not applicable since both posterior obliques are needed to capture targets posterior of the metal dental fillings and we do not use a single beam to deliver full prescription to any part of the target. A variant 5-beam technique is therefore used ~~in this case.~~ where ~~A~~all four oblique beams ~~are allowed to~~can place spots across the midline unless otherwise blocked by their respective SAVs ~~(such as those derived from metals)~~. An~~a~~ SFO section on the ipsilateral side is defined within each of the posterior oblique beam's SPV, as seen in Fig. 2(b) to (d). The

dosimetric goal is again to have the posterior oblique beams deliver half of the prescribed dose uniformly in a SFO-like manner to the ipsilateral side of the target; and the other half of the prescription ~~is~~ delivered as MFO from the other three oblique beams ~~for the contralateral side use a MFO combination to deliver the other half of the prescription~~ to achieve ~~the~~ desired OAR sparing. ~~To achieve this, a SFO section on the ipsilateral side is defined within each of the posterior oblique beam's SPV, as seen in Fig. 2(b) to (d).~~ Also seen in Fig. 2(d) is a 2cm wide 'control' region (blue) separating the two SFO sections (green and red) of the posterior oblique beams. Like the transition region (orange) in Fig. 1(a) to (d), this control region permits proper dose gradients for the two uniform dose distributions from the SFO sections and avoids dose heterogeneity at the junction due to setup, range, and anatomical uncertainties.

Dosimetric and Robustness evaluations

For comparison, the second bilateral head and neck case with sinus involvement was re-planned using two additional MFO techniques: conventional MFO (MFOcon) without any SPV or SAV volumes and MFO with only SPV volumes (MFOspv). These additional MFO techniques are ~~more~~ commonly used in PBS treatment planning for head and neck cancers. ~~The purpose of this comparison is to determine if there are any obvious advantages in plan robustness and OAR sparing in the MFOspv/sav method presented.~~ All plans used the same five beams and robustness settings. The optimization objectives on the original target and OAR contours are identical between the MFOcon, MFOspv, and MFOspv/sav plans. ~~Extra segmented target contours were needed for MFOspv and MFOspv/sav to control spot placement. Additional uniform dose objectives on further segmented targets were needed to control dose in the SFO-like regions of the MFOspv/sav plan.~~ Detailed planning ~~s of these~~ objectives are included in Supplemental Table S1. All three MFO plans are normalized such that 97% of the CTV63 volume is covered by prescription, i.e. D97% = 63 Gy(RBE). These three plans are then designated as the nominal plans in Tables 1 and 2.

Plan robustness for these three MFO plans ~~, MFOcon, MFOspv, and MFOspv/sav,~~ were evaluated using both simulated uncertainty scenarios and forward-calculated plans on VFCTs taken over the course of the patient's treatment. The robustness evaluation shown in Table 1 and Fig. S1 is part of the standard physics check for all patients before treatment starts. For each uncertainty scenario shown in Table 1, robustness of these three MFO plans was evaluated by deliberately shifting the location of the isocenter by +/-0.3 cm in the x, y and z directions to simulate setup errors, together with +/-4% density perturbation to account for range uncertainties. For example, Scenario 1 corresponds to an isocenter shift of 3mm to the right, 3mm to the anterior, and 3mm to the inferior, as well as a 4% over-range in HU-to-stopping power calibration. A total of 16 different scenarios are evaluated.

Weekly VFCTs were taken during the treatment course. These VFCTs were registered to the planning CT, and the various target and OAR structures were transferred to the VFCTs and

reviewed by the attending physician. The three nominal MFO plans, MFOcon, MFOspv, and MFOspv/sav, were then forward-calculated on these VFCTs for inter-fractional robustness evaluation. The results are summarized in Table 2 and Fig. S2. Values that do not meet our clinical criteria are highlighted in yellow.

Results

The 4-beam arrangement

Dose distributions for the 4-beam technique are shown in Fig. 3. As seen in Fig. 3(a, b), the uniform dose objectives ensured that the AP and PA beams delivered a uniform, i.e. SFO-like, dose distribution to their respective SFO structures within each SPV (excluding the transition region). Note that the PA beam's dose distribution shows three target dose levels (CTV56, CTV63, and CTV70) since this part of the target has simultaneous integrated boost. The AP beam on the other hand treats the inferior nodes and has only one target dose level (CTV56). Note that SFO structures must be created separately for each dose level. A uniform dose objective set to the overall PTV helps ensure overall dose homogeneity and guides the LAO and RAO beams to deliver the remaining half of the prescription using the uniform dose distributions from AP and PA as a baseline. This results in This optimization setting ensures that the anterior oblique beams produceing SFO-like dose distributions in the inferior neck when it their SPVs overlaps only with either the AP or PA beam's SFO regions; and at the same time it also allows MFO like dose distributions in the superior neck for better OAR sparing superiorly where the SPVs of the anterior obliques overlap (Fig. 3(c) to (h)). This beam arrangement prevents dose spillage across the midline in the inferior neck and avoids entering the parotids in the superior neck for maximum OAR sparing.

The 5-beam arrangements: standard and variant

Dose distributions for the 5-beam techniques are shown in Fig. 4. Unlike the 4-beam technique, the posterior oblique beams in the 5-beam arrangement are also used to treat around the spinal cord to the contralateral side behind the contralateral parotids. The posterior oblique beams' SAVs don't include a posterior extension of the spinal cord (and brainstem if applicable).
As demonstrated in Fig. 4(a) to (d) using standard 5-beam technique, at levels where the target volume can be separated into disjoint left and right segments, the segment on the left receives half of the prescription uniformly (i.e. SFO-like) from the LPO beam, and the other half of the prescribed dose is contributed by an MFO combination from the RPO and LAO beams. The contralateral anterior oblique beam, i.e. RAO here, does not contribute to the target on the left in this case. The same is true for the target segment on the right. In the sinus region, the AP beam is used to deliver half of the prescription uniformly to its SFO structure, and the remaining dose is contributed in MFO dose distributions by the other four obliques.

As seen in Fig. 4(e) to (h), at levels where the target volume cannot be separated into disjoint left and right segments, the variant 5-beam arrangement again splits the prescription in two halves, i.e. using the posterior oblique beams to deliver the first half in uniform, SFO-like dose distributions to the ipsilateral targets, and simultaneously allows MFO dose contributions from the other three oblique beams (i.e. including the contralateral anterior oblique beams as long as they are not blocked by their respective SAVs due to metal dental fillings) to deliver the other half of the prescription for better OAR sparing.

Dosimetric and robustness evaluations

The MFOspv/sav plan shows superior OAR sparing over both MFOcon and MFOspv plans. Table 1 shows that while all three nominal plans satisfy the physician's requirement on target coverage and OAR dose limits, the MFOcon plan has only two OARs with the lowest dose values out of the total 14 OARs (chiasm and left temporal lobe), the MFOspv plan has three (cord, left submandibular gland, and right temporal lobe), and the MFOspv/sav has nine. This demonstrated the superiority in OAR sparing for MFOspv/sav.

Robustness evaluations using both simulated scenarios and forward-calculated plans on the weekly VFCT scans confirmed ~~the superior~~ plan robustness for the MFOspv/sav plan. Among all simulated scenarios seen in Table1 and Fig. S1 only four of the dose statistics values failed our clinical criteria for the MFOspv/sav, while a total of 17 and 20 dose statistics values failed for the MFOspv and the MFOcon plans, respectively. The averaged overall D1.0cc (Table 1, last column) of the simulated scenarios are 114.5%, 109.6%, and 112.7% of Rx MFOcon, MFOspv and MFOspv/sav, respectively, and all are within our clinical criteria of 115%. The CTV coverage requirement for the nominal plan is D97%>99% and the robustness requirement is that the averaged coverage from all 16 scenarios achieves D97%>94%. All methods meet this minimum CTV coverage requirement.

For forward-calculated VFCT plans, as seen in Table2 and Fig. S2, target coverages are largely conserved for all MFO plans across all 7 VFCTs, with the averaged CTV56 D97% coverage at 55.9, 55.7, and 55.9 Gy(RBE), and CTV63 D97% coverage at 62.5, 63.0, and 62.9 Gy(RBE) for MFOspv/sav, MFOspv, and MFOcon, respectively. In addition, dose values for 12 of the 14 OARs are also relatively unchanged from those of their respective nominal plans for all 7 VFCTs. Specifically, the 9 OARs (brainstem, parotids, lacrimal glands, larynx, oral cavity, and cochleae) for which the nominal MFOspv/sav plan has the lowest values, all continue to be consistently the lowest among all three MFO plans for all VFCTs. The cord maximum dose (Dmax), on the other hand, continues to be the lowest for the MFOspv plan, although all three MFO plans consistently achieved Dmax less than 40 Gy(RBE) in all VFCTs. Largest dose variations for all three MFO plans are seen in the optic chiasm's D0.05cc values due to proximity to the target and shape of the OAR. The MFOspv/sav plan still has an advantage for the optic chiasm where its D0.05cc vales are less than 54 Gy(RBE) in two of the seven VFCTs,

while the MFOcon plan has one and the MFOspv plan has all of its chiasm D0.05cc values over 54 Gy(RBE) amongst all VFCTs.

Discussions

The 5-beam technique typically achieves excellent parotid sparing and provides better sparing for medial OARs such as the oral cavity and pharyngeal constrictor. In addition, the versatility of the 5-beam arrangement to adapt to different target anatomies also broadens its use. As a result, almost all bilateral H&N cases are treated with this 5-beam arrangement in our clinics. Specifically, spot guidance structures and associated planning objectives at levels with (Fig. 2(b) to (d)) and without (Fig. 2(a)) medial involvement follow the variant and the standard 5-beam techniques, respectively. The resultant dose distribution at levels with and without medial involvement therefore resembles that of the variant (Fig. 4(e) to (h)) and the standard (Fig. 4 (a) to (d)) 5-beam arrangement, respectively. Note that with the 5-beam arrangement, cord maximum dose typically is not the limiting factor and as a result, the SAVs for the posterior obliques are often edited to allow better parotid sparing.

The SFO regions of the AP and PA beams have a CSI-like gradient dose matching in their 'transition' region (orange in Fig. 1). The 'control' region (blue in Fig. 2(d)) separating the respective SFO-regions of the LPO and RPO in the midline where the target is connected medially act~~s~~ in the same manner. This CSI-like dose gradient effectively mitigates the potential dose heterogeneity when changes in the day-to-day setup cause beams to "bump into" or "be separated from" each other. Indeed, such dose gradients have successfully mitigated overlaps and/or separations, and reduced hot/cold spots for proton CSI treatments. In addition, ~~perhaps more importantly,~~ the MFOspv/sav technique's integration of SFO regions also specifically limits each individual beam's contribution to any part of the target to half of the prescription. In our experience, when no part of the target is relying on one single beam to deliver the majority of the prescription, the resulting plan is less likely to show large magnitude heterogeneity in forward-calculated VFCT plans.

The three MFO planning techniques presented here, i.e. MFOcon, MFOspv, and MFOspv/sav, each relies progressively more on user-imposed guidance on top of the optimization process driven by the cost function. The resulting solution spaces for these three MFO techniques therefore shrink from MFOcon to MFOspv, and then again from MFOspv to MFOspv/sav. As a result, in theory, with a larger solution space the MFOcon technique indeed does not prevent the TPS from finding the same optimization result as the MFOspv and MFOspv/sav techniques. In practice, however, we have not yet encountered an instance where dose statistics and plan robustness of the MFOspv/sav plan can be achieved by simply using the MFOspv or MFOcon. We surmise that this is because current TPS optimizers do not have a consistent method to reach the desired local minimum without specific user guidance.

Potentially one can optimize with robust Dmax constraints for the OARs in all three MFO approaches. However, in our experience, such setting typically results in a much lower OAR Dmax and under-coverage of the CTV in the nominal plan. Iterative adjustment is needed to find the "right" robustness setting. In addition, this iterative trial-and-error process became intractable when one needs to manage multiple critical OARs while simultaneously maintaining CTV coverage. The MFOspv/sav method reaches directly the achievable minimum OAR values with the desired CTV coverage without relying on robust Dmax settings.

The robustness observed in all MFO plans in the VFCT evaluations is a result of the setup and range margins added during robust optimization. To include anatomical variations into robust optimization would require *a priori* model to predict patients' interfractional anatomical changes. Further studies are needed ~~in order~~ to disentangle the effect of setup errors and range uncertainties from that of anatomical changes.

The MFOspv/sav technique is a systematic approach utilizing SPV and SAV to guide the optimizer to consistently reach ~~optimal~~ desired OAR dose values and plan robustness. This results in a more efficient planning process with fewer optimizations required to reach the desired dose distribution and less reliance on user experience which can result in inconsistencies in the resulting plan ~~quality~~. ~~Overall, a~~As the current TPS still cannot automatically optimize beam angles and spot placements together with spot weights, MFOspv/sav's guidance on spot placements based on anatomy and beam angles leads the optimizer to more consistent plan quality.

Figure 1: View of (a) AP, (b) PA, (c) RAO and (d) LAO spot placement volumes (yellow, blue, green, and pink, respectively) with the transition region in orange. The SPV for LAO is in pink shade and RAO in green outline (e, f). Both RAO and LAO share the same SAV (in blue outline). The SPVs for both RAO and LAO are connected (e) across the midline and separated (f) below the parotids.

Figure 2: (a) Beam arrangement for the 5-beam techniques. SAVs are shown in green. (b) SPV (pink) and SAV (green) for the LPO. (c) SPV (pink) and SAV (orange) for the RPO beam. (d) SFO sections of RPO and LPO are separated by a 2 cm gap at midline. The SFO section of the RPO (green) and the SFO section of the LPO (red) are seen to be separated by a 2 cm control region (blue).

Figure 3: Dose distributions of the AP (a) and PA (b) beams (coronal), and the RAO (c, f), PA (b, d) and LAO (e, h) beams (axial) for the 4-beam technique. For the superior portion of the target (c, d, e), half of the prescription is delivered through the PA beam using a uniform dose criterion. Only AP, RAO and LAO are used for the inferior portion of the target (f, g, h).

Figure 4: Dose distributions of (a) RPO, (b) RAO, (c) LAO and (d) LPO beams for the 5-beam technique at levels where the target can be separated into left and right segments. Dose distributions from the (e) RPO, (f) RAO, (g) LPO and (h) LAO beams for the variant 5-beam technique at levels where the target has medial involvement.

Table 1: Robustness evaluation of the three MFO planning techniques using simulated scenarios.

Table 2: Inter-fraction plan robustness evaluation of the three MFO planning techniques using VFCTs taken weekly over the treatment course.

1. P. Blanchard *et al.*, Proton Therapy for Head and Neck Cancers. *Semin Radiat Oncol* **28**, 53-63 (2018).
2. A. C. Moreno *et al.*, Intensity modulated proton therapy (IMPT) - The future of IMRT for head and neck cancer. *Oral Oncol* **88**, 66-74 (2019).
3. X. Li, A. Lee, M. A. Cohen, E. J. Sherman, N. Y. Lee, Past, present and future of proton therapy for head and neck cancer. *Oral Oncol* **110**, 104879 (2020).
4. P. DeLuca Jr, A. Wambersie, G. Whitmore, ICRU Report 78: Prescribing, Recording, and Reporting Proton-Beam Therapy. *J ICRU* **7**, 1-210 (2007).
5. H. Paganetti, Range uncertainties in proton therapy and the role of Monte Carlo simulations. *Phys Med Biol* **57**, R99-117 (2012).
6. J. Unkelbach *et al.*, Robust radiotherapy planning. *Phys Med Biol* **63**, 22TR02 (2018).
7. A. J. Lomax, Myths and realities of range uncertainty. *Br J Radiol* **93**, 20190582 (2020).
8. K. Langen, M. Zhu, Concepts of PTV and Robustness in Passively Scattered and Pencil Beam Scanning Proton Therapy. *Semin Radiat Oncol* **28**, 248-255 (2018).
9. R. Mohan, I. J. Das, C. C. Ling, Empowering Intensity Modulated Proton Therapy Through Physics and Technology: An Overview. *Int J Radiat Oncol Biol Phys* **99**, 304-316 (2017).
10. A. J. Lomax *et al.*, Intensity modulated proton therapy: a clinical example. *Med Phys* **28**, 317-324 (2001).
11. W. Liu, X. Zhang, Y. Li, R. Mohan, Robust optimization of intensity modulated proton therapy. *Med Phys* **39**, 1079-1091 (2012).
12. W. Gu *et al.*, Robust optimization for intensity-modulated proton therapy with soft spot sensitivity regularization. *Med Phys* **46**, 1408-1425 (2019).
13. J. Ma *et al.*, Robust radiobiological optimization of ion beam therapy utilizing Monte Carlo and microdosimetric kinetic model. *Phys Med Biol* **65**, 155020 (2020).
14. S. J. Frank *et al.*, Multifield optimization intensity modulated proton therapy for head and neck tumors: a translation to practice. *Int J Radiat Oncol Biol Phys* **89**, 846-853 (2014).
15. A. Anand, M. Bues, M. E. Gamez, C. Stefan, S. H. Patel, Individual Field Simultaneous Optimization (IFSO) in spot scanning proton therapy of head and neck cancers. *Med Dosim* **44**, 375-378 (2019).
16. K. H. Kang *et al.*, Proton therapy for head and neck paragangliomas: A single institutional experience. *Head Neck* **42**, 670-677 (2020).
17. A. Bolsi *et al.*, Pencil beam scanning proton therapy for the treatment of craniopharyngioma complicated with radiation-induced cerebral vasculopathies: A dosimetric and linear energy transfer (LET) evaluation. *Radiother Oncol* **149**, 197-204 (2020).
18. M. J. Pelak *et al.*, Clinical outcomes of head and neck adenoid cystic carcinoma patients treated with pencil beam-scanning proton therapy. *Oral Oncol* **107**, 104752 (2020).
19. M. Yoon *et al.*, Craniospinal irradiation techniques: a dosimetric comparison of proton beams with standard and advanced photon radiotherapy. *Int J Radiat Oncol Biol Phys* **81**, 637-646 (2011).
20. U. Mock, D. Georg, J. Bogner, T. Auberger, R. Pötter, Treatment planning comparison of conventional, 3D conformal, and intensity-modulated photon (IMRT) and proton therapy for paranasal sinus carcinoma. *Int J Radiat Oncol Biol Phys* **58**, 147-154 (2004).
21. A. J. Lomax, E. Pedroni, H. Rutz, G. Goitein, The clinical potential of intensity modulated proton therapy. *Z Med Phys* **14**, 147-152 (2004).
22. L. Cozzi, A. Fogliata, A. Lomax, A. Bolsi, A treatment planning comparison of 3D conformal therapy, intensity modulated photon therapy and proton therapy for treatment of advanced head and neck tumours. *Radiother Oncol* **61**, 287-297 (2001).

23. K. Stützer, A. Lin, M. Kirk, L. Lin, Superiority in Robustness of Multifield Optimization Over Single-Field Optimization for Pencil-Beam Proton Therapy for Oropharynx Carcinoma: An Enhanced Robustness Analysis. *Int J Radiat Oncol Biol Phys* **99**, 738-749 (2017).
24. A. Beddok *et al.*, Proton therapy for head and neck squamous cell carcinomas: A review of the physical and clinical challenges. *Radiother Oncol* **147**, 30-39 (2020).
25. A. S. Grewal, J. Jones, A. Lin, Palliative Radiation Therapy for Head and Neck Cancers. *Int J Radiat Oncol Biol Phys* **105**, 254-266 (2019).
26. A. Lee *et al.*, Trends and Disparities of Proton Therapy Use among Patients with Head and Neck Cancer: Analysis from the National Cancer Database (2005-14). *Int J Part Ther* **5**, 1-10 (2019).
27. J. E. Leeman *et al.*, Proton therapy for head and neck cancer: expanding the therapeutic window. *Lancet Oncol* **18**, e254-e265 (2017).
28. G. B. Gunn *et al.*, Clinical Outcomes and Patterns of Disease Recurrence After Intensity Modulated Proton Therapy for Oropharyngeal Squamous Carcinoma. *Int J Radiat Oncol Biol Phys* **95**, 360-367 (2016).
29. A. J. Lomax, M. Goitein, J. Adams, Intensity modulation in radiotherapy: photons versus protons in the paranasal sinus. *Radiother Oncol* **66**, 11-18 (2003).
30. H. Lin *et al.*, Supine craniospinal irradiation using a proton pencil beam scanning technique without match line changes for field junctions. *Int J Radiat Oncol Biol Phys* **90**, 71-78 (2014).

Figure2

Click here to access/download;Figure;Figures_Revised_2021.pdf

Fig. 1

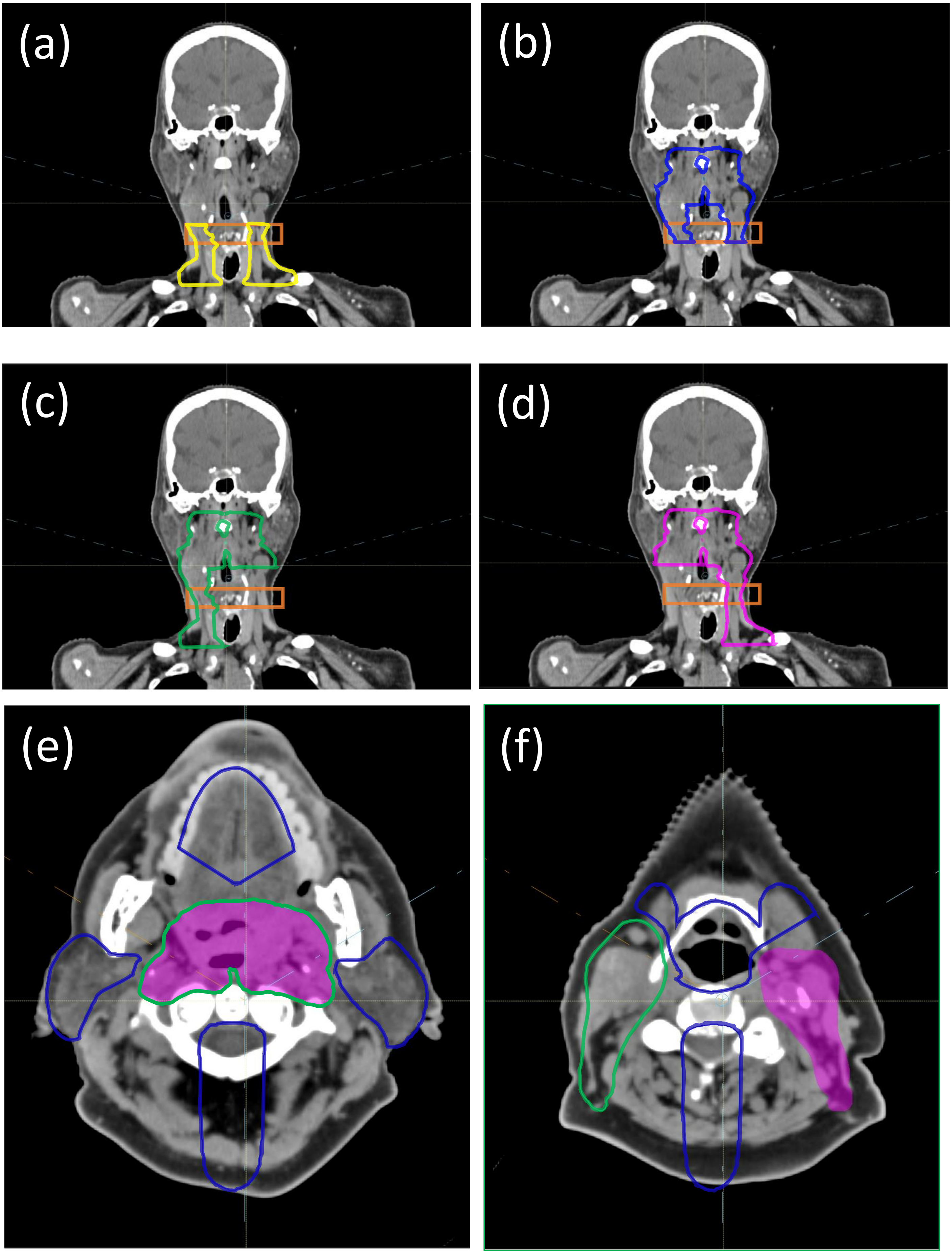

Fig. 2

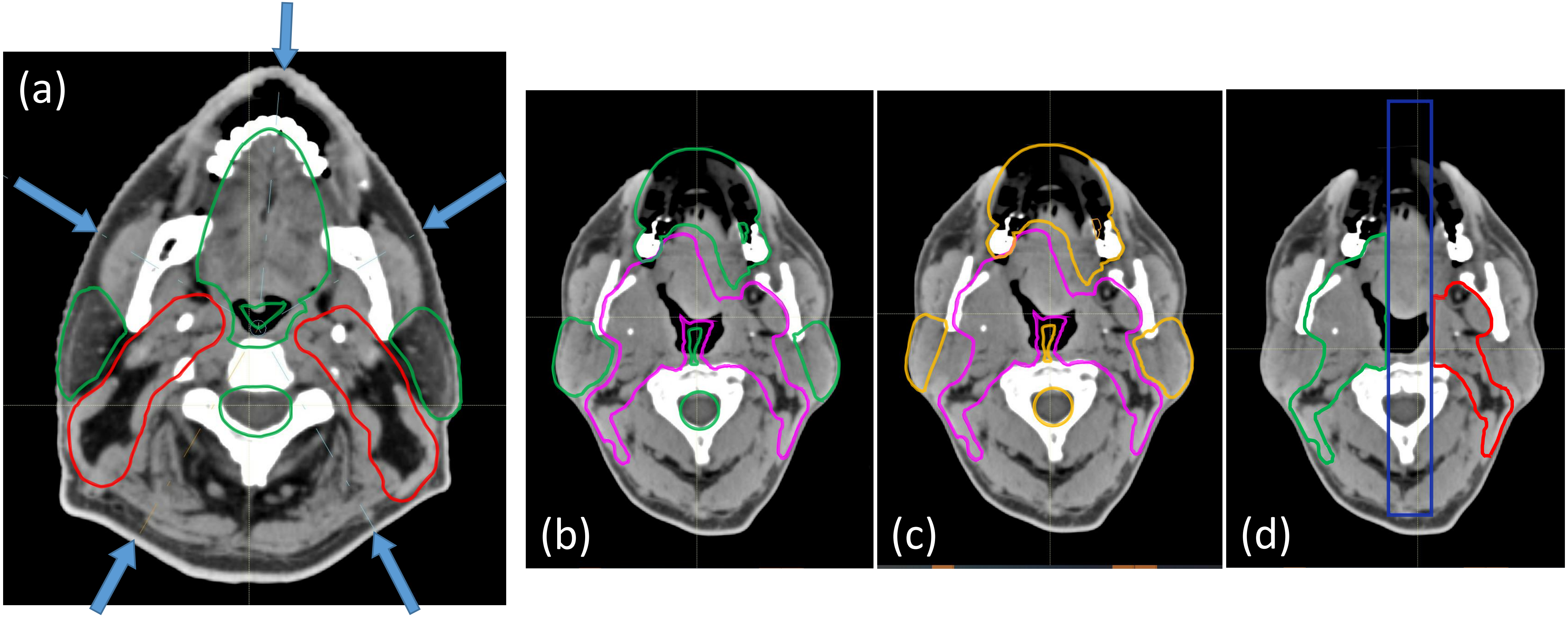

Fig. 3

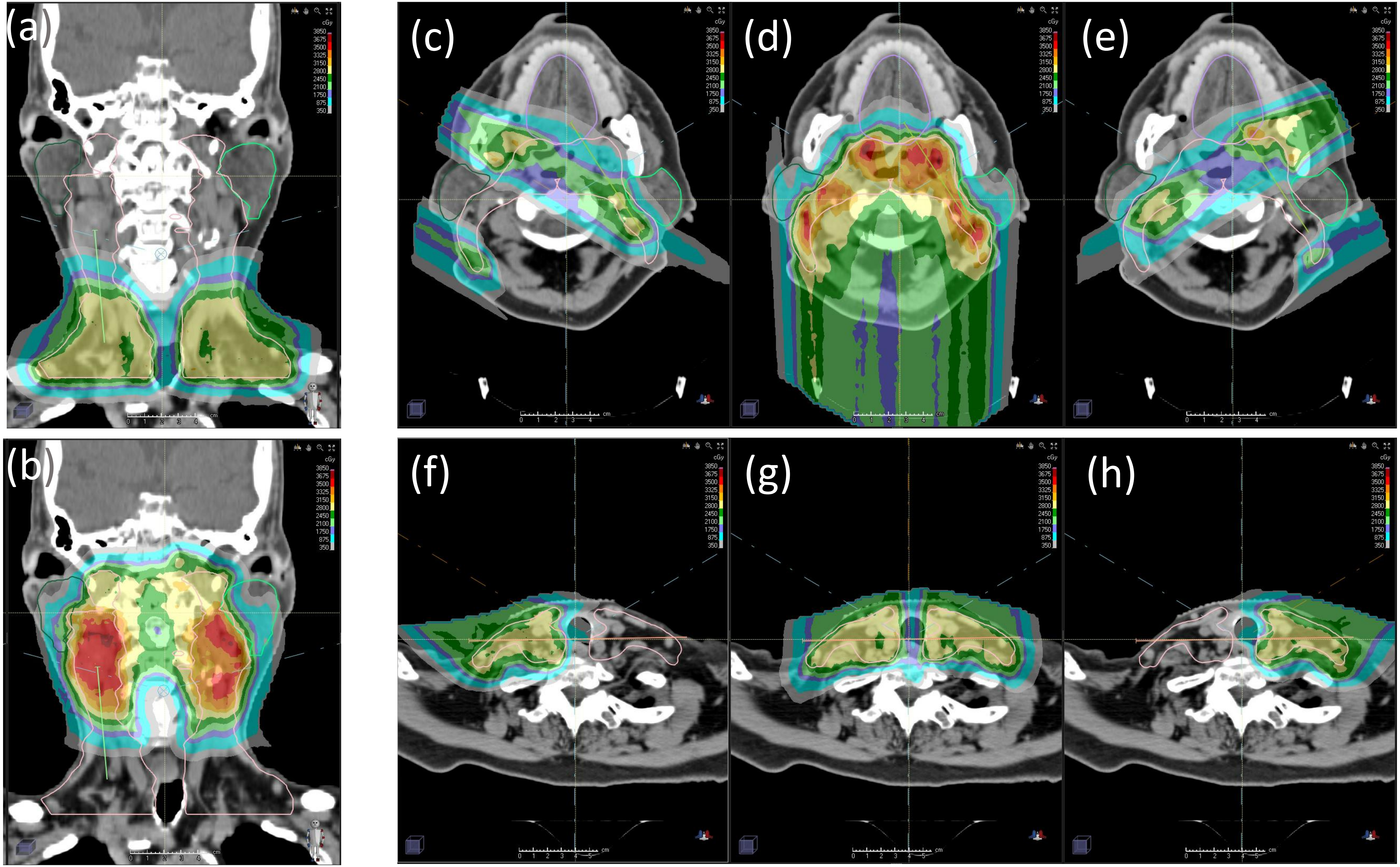

Fig. 4

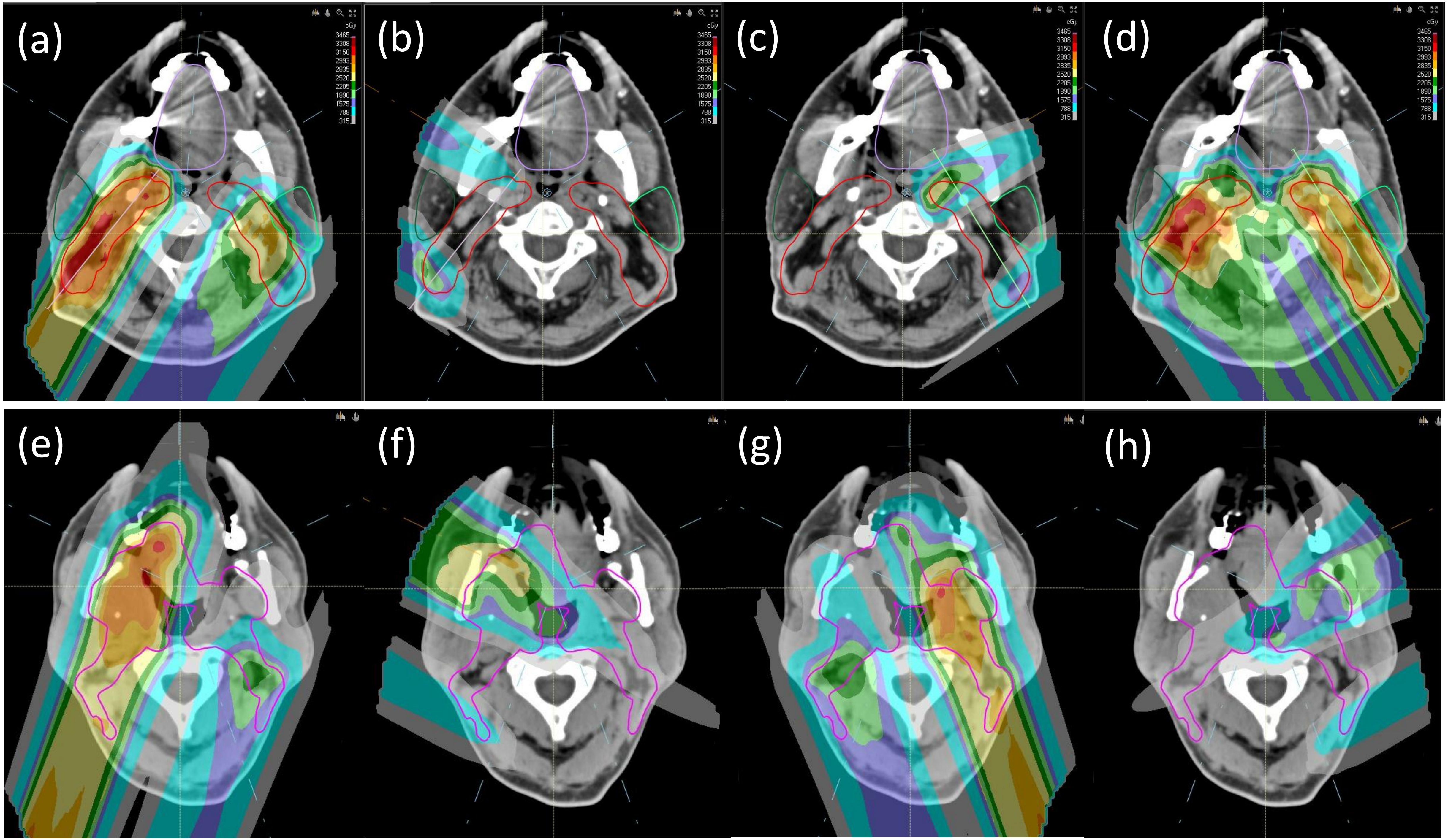
(a)
(b)
(c)
(d)
(e)
(f)
(g)
(h)
cGy
3465
3308
3150
2993
2835
2520
2205
1890
1575
788
315

Table 1

		CTV63 D97%	CTV56 D97%	Cord Dmax	Chiasm D0.05cc	Brainstem Dmax	Parotid Left Dmean	Parotid Right Dmean	Lacrimal Gland Left Dmean	Lacrimal Gland Right Dmean	Submendibular Gland Left Dmean	Larynx Dmean	Oral Cavity Dmean	Cochlea Left Dmax	Cochlea Right Dmax	Temporal Lobe Left D2.0cc	Temporal Lobe Right D2.0cc	External D1.0cc
MFO using SPV and SAV	Nominal	63.2	56.4	35.6	46.3	45.5	11.8	12.6	17.0	16.8	29.1	14.6	9.2	14.4	16.1	42.5	42.1	67.4
	Scenario 1	60.6	54.4	41.2	41.0	40.8	9.6	17.0	18.6	27.1	36.0	19.7	13.0	10.5	11.3	34.8	45.8	73.2
	Scenario 2	61.6	54.7	39.5	51.8	47.7	11.8	20.3	14.7	19.2	34.5	18.4	9.1	16.9	26.1	39.6	53.8	72.7
	Scenario 3	61.0	52.6	35.9	45.4	54.4	9.6	16.5	14.6	25.3	29.4	18.1	11.5	13.4	15.7	38.5	45.7	72.9
	Scenario 4	61.1	52.4	35.3	55.1	61.9	11.6	20.0	10.6	16.3	28.3	17.5	7.8	24.9	33.1	42.6	53.9	71.7
	Scenario 5	60.0	54.6	34.9	39.7	41.0	16.3	10.8	25.9	18.2	42.9	19.9	15.1	10.0	10.5	42.8	33.2	73.1
	Scenario 6	61.2	54.8	31.2	51.4	47.4	19.2	13.9	21.1	13.2	41.9	18.3	11.0	22.9	24.2	49.7	40.9	70.6
	Scenario 7	58.9	53.6	35.4	43.6	50.9	16.2	10.5	22.1	17.5	36.3	19.1	13.3	13.7	13.5	46.5	34.8	73.6
	Scenario 8	58.9	53.6	34.0	53.5	57.7	19.1	13.4	17.4	10.9	35.1	18.1	9.5	29.2	30.3	52.5	42.4	71.9
	Scenario 9	59.1	52.3	42.7	38.9	35.9	7.3	12.9	17.2	24.1	22.7	13.8	10.2	10.2	8.8	34.0	40.7	70.1
	Scenario 10	59.5	53.4	40.2	50.7	40.2	9.2	15.9	13.4	17.0	21.1	11.9	6.3	14.2	17.0	38.8	49.4	69.6
	Scenario 11	59.9	51.5	36.8	42.8	46.2	6.7	12.3	13.0	22.0	16.3	11.9	8.7	12.6	12.3	38.2	41.2	69.7
	Scenario 12	58.8	51.9	36.4	53.3	52.2	8.2	15.4	9.1	14.0	14.9	10.8	5.2	22.2	23.8	42.2	49.7	69.3
	Scenario 13	57.7	53.6	36.4	35.0	34.7	13.0	8.0	23.4	16.2	31.0	14.5	11.7	9.0	9.1	40.7	30.5	69.7
	Scenario 14	58.7	54.8	33.0	49.8	39.9	15.4	10.8	18.5	11.6	29.5	12.9	7.5	18.5	15.8	48.0	37.5	69.1
	Scenario 15	56.7	52.9	36.9	38.6	41.3	12.8	7.2	19.4	15.7	24.7	13.7	10.1	12.8	10.8	44.8	32.6	69.2
	Scenario 16	56.2	53.9	34.7	50.9	50.3	15.0	9.7	14.7	9.4	23.1	12.5	6.3	26.0	22.2	50.9	39.3	69.7
MFO using SPV only	Nominal	63.3	56.2	31.6	46.5	53.2	20.6	21.3	21.7	21.0	28.0	20.6	13.9	17.4	19.5	36.9	40.3	67.1
	Scenario 1	62.4	54.7	30.7	43.5	45.9	16.9	24.3	19.1	26.3	37.4	25.6	18.3	11.7	16.3	28.3	40.8	71.1
	Scenario 2	62.3	54.5	30.7	54.1	53.3	20.6	27.7	20.0	25.7	36.3	22.9	13.9	18.2	25.6	35.7	52.5	70.1
	Scenario 3	61.7	53.2	32.9	47.7	55.0	16.4	24.1	19.0	27.2	30.7	22.1	15.8	13.7	18.7	31.7	43.1	70.4
	Scenario 4	61.9	52.1	32.7	56.0	62.5	19.9	27.3	19.0	24.5	29.7	19.9	11.7	22.6	29.9	38.5	53.9	69.2
	Scenario 5	60.6	54.8	30.2	41.2	43.5	23.0	18.5	26.8	17.4	39.6	27.0	19.8	13.5	13.2	38.3	29.8	70.0
	Scenario 6	61.3	55.1	29.5	52.8	54.3	27.2	22.0	25.5	19.0	38.5	24.0	15.4	22.8	20.2	46.6	40.4	69.5
	Scenario 7	60.1	53.3	32.8	48.1	53.5	22.5	18.4	26.2	19.6	32.1	24.2	17.3	16.3	14.7	42.5	31.8	69.6
	Scenario 8	60.0	52.8	30.9	56.1	62.9	26.4	21.4	24.8	19.5	31.1	21.5	13.2	28.1	24.9	49.8	41.7	68.5
	Scenario 9	61.3	53.0	31.6	41.2	43.2	15.7	21.7	17.4	22.4	26.1	21.9	15.5	11.4	16.0	24.4	36.2	69.1
	Scenario 10	60.6	53.3	32.0	52.0	50.4	19.4	25.3	18.2	22.9	24.9	18.9	11.2	16.1	23.6	31.2	48.3	68.6
	Scenario 11	59.9	52.8	33.7	43.0	50.3	15.0	21.4	17.3	23.3	18.9	18.4	13.3	13.8	18.7	28.2	38.4	68.8
	Scenario 12	59.9	53.2	33.7	53.2	57.5	18.3	24.7	17.3	21.6	17.7	15.9	9.3	21.0	28.4	34.1	49.0	68.2
	Scenario 13	59.1	54.3	29.9	37.0	38.1	21.5	16.6	23.9	15.0	27.8	23.6	16.9	13.2	12.8	34.0	25.7	68.2
	Scenario 14	59.0	55.0	29.7	48.4	50.1	25.5	20.2	22.6	17.2	26.5	20.5	12.5	20.7	18.8	41.9	36.0	68.4
	Scenario 15	58.6	53.0	32.1	39.8	47.5	21.1	16.1	23.4	17.4	20.4	20.8	14.7	16.1	14.4	38.0	28.2	67.9
	Scenario 16	58.3	54.2	30.8	49.0	58.3	24.7	19.3	21.7	17.6	19.2	18.0	10.7	26.2	23.9	44.6	37.8	67.5
Conventional MFO	Nominal	63.3	56.5	32.4	45.0	50.5	21.0	21.7	25.1	22.1	28.3	20.7	21.5	16.2	18.4	36.7	41.3	68.5
	Scenario 1	61.8	54.7	33.0	41.1	44.5	17.5	25.6	21.0	26.1	36.5	23.8	23.9	10.3	15.6	26.7	42.9	75.2
	Scenario 2	62.0	54.9	32.5	56.8	51.6	22.0	28.5	24.1	25.5	35.2	22.8	21.1	17.4	23.4	35.0	53.7	76.2
	Scenario 3	61.3	53.8	31.6	45.1	53.3	16.5	24.4	23.2	28.0	28.4	22.3	21.6	12.8	18.3	32.3	45.3	75.2
	Scenario 4	61.6	53.9	31.3	55.8	61.9	20.8	26.8	25.7	25.5	26.9	21.3	19.0	23.2	29.3	39.5	54.5	74.7
	Scenario 5	61.5	54.9	33.6	40.2	42.0	23.1	19.5	27.8	17.8	39.4	25.4	25.8	11.9	13.1	36.8	32.1	73.0
	Scenario 6	61.3	55.5	32.6	53.6	50.9	28.2	22.0	29.3	19.1	38.7	24.1	23.1	21.8	19.0	46.2	41.9	73.0
	Scenario 7	60.7	54.5	32.0	45.7	54.0	22.1	18.2	28.5	20.5	30.6	24.8	23.4	15.1	14.5	42.4	34.0	73.4
	Scenario 8	60.4	55.1	31.6	55.1	61.6	26.9	20.4	29.9	20.6	29.6	23.2	20.9	27.3	24.2	50.5	43.2	71.4
	Scenario 9	60.7	52.9	34.1	35.9	41.2	16.0	23.4	18.3	23.9	29.5	20.4	22.9	10.4	15.2	23.7	37.3	71.0
	Scenario 10	59.7	52.6	33.3	50.5	47.0	20.2	26.2	21.8	23.6	27.9	19.2	20.1	16.3	21.2	31.1	48.6	71.9
	Scenario 11	58.7	52.5	32.8	38.0	47.1	15.2	22.3	20.8	25.8	21.0	19.3	20.7	12.3	17.5	29.4	39.3	71.4
	Scenario 12	58.8	52.6	32.2	49.3	56.1	19.0	24.6	23.7	23.9	19.2	17.9	18.2	21.8	27.5	36.0	49.0	69.8
	Scenario 13	59.1	54.0	34.3	34.5	35.8	21.4	17.7	24.1	16.6	32.2	22.3	24.7	12.2	12.3	33.2	27.2	70.2
	Scenario 14	58.2	55.1	33.8	48.6	46.5	26.1	20.1	26.1	18.2	31.1	20.8	22.0	20.9	17.5	41.4	36.8	70.2
	Scenario 15	58.0	53.3	33.3	40.1	46.1	20.6	16.5	25.3	19.4	23.4	21.9	22.4	15.0	14.3	38.5	29.6	69.3
	Scenario 16	57.6	54.5	32.3	51.4	55.8	25.0	18.5	27.0	19.7	22.1	20.1	19.9	26.7	23.0	45.2	38.5	68.8

Table 2

		CTV63 D97%	CTV56 D97%	Cord Dmax	Chiasm D0.05cc	Brainstem Dmax	Parotid Left Dmean	Parotid Right Dmean	Lacrimal Gland Left Dmean	Lacrimal Gland Right Dmean	Submendibular Gland Left Dmean	Larynx Dmean	Oral Cavity Dmean	Cochlea Left Dmax	Cochlea Right Dmax	Temporal Lobe Left D2.0cc	Temporal Lobe Right D2.0cc	External D1.0cc
MFO using SPV and SAV	Nominal	63.2	56.4	35.6	46.3	45.5	11.8	12.6	17.0	16.8	29.1	14.6	9.2	8.2	8.2	42.5	42.1	67.4
	VFCT 1	62.2	55.1	33.8	53.8	48.1	12.6	13.6	16.2	19.6	25.9	12.7	11.1	9.4	8.5	40.7	38.4	69.4
	VFCT 2	62.8	56.2	32.6	55.3	46.3	14.0	12.0	16.2	17.8	30.3	14.2	9.7	8.5	8.5	41.2	39.8	68.0
	VFCT 3	62.7	56.1	32.4	55.3	47.3	14.5	12.1	14.1	18.1	33.3	15.5	10.0	8.3	8.5	42.3	42.8	68.3
	VFCT 4	62.2	56.0	35.4	53.5	42.9	11.8	13.5	18.7	18.0	31.5	16.9	10.6	8.3	8.9	41.2	42.8	68.3
	VFCT 5	62.7	56.0	35.2	54.4	47.7	13.6	13.1	16.7	16.8	34.5	17.0	10.0	8.5	9.5	42.4	44.1	69.5
	VFCT 6	62.5	55.8	32.7	54.7	45.1	14.0	12.9	16.1	16.1	35.3	18.3	12.4	8.8	10.6	42.5	45.3	69.4
	VFCT 7	62.2	55.7	35.0	56.6	48.2	14.8	14.1	14.8	15.5	37.8	21.3	12.9	9.0	10.2	43.1	44.6	70.6
MFO using SPV only	Nominal	63.3	56.2	31.6	46.5	53.2	20.6	21.3	21.7	21.0	28.0	20.6	13.9	17.4	19.5	36.9	40.3	67.1
	VFCT 1	62.4	54.9	31.0	55.7	52.9	20.9	22.0	20.6	21.0	29.2	19.4	15.6	12.0	12.2	33.9	37.0	68.1
	VFCT 2	63.0	55.9	30.6	58.6	54.2	22.4	20.7	20.6	21.6	29.3	21.2	14.2	12.2	11.9	35.6	38.8	67.8
	VFCT 3	63.3	55.9	30.6	58.9	54.1	22.8	20.7	19.8	23.0	30.6	22.7	14.6	12.2	11.6	37.1	41.8	67.8
	VFCT 4	63.1	55.9	31.3	55.3	53.1	20.2	22.4	22.7	20.9	28.5	23.5	14.7	11.4	13.0	36.0	40.0	68.7
	VFCT 5	63.3	56.0	31.2	57.9	51.5	21.9	21.6	22.2	21.2	31.1	24.0	14.3	12.0	12.5	38.1	41.8	68.3
	VFCT 6	63.1	55.8	31.4	57.6	53.4	22.6	21.5	21.4	21.3	31.7	23.7	15.5	12.4	13.1	37.8	42.2	69.2
	VFCT 7	62.8	55.7	32.4	57.4	54.4	22.2	22.1	21.8	21.6	33.9	27.6	16.0	11.9	12.2	38.3	41.1	69.0
Conventional MFO	Nominal	63.3	56.5	32.4	45.0	50.5	21.0	21.7	25.1	22.1	28.3	20.7	21.5	16.2	18.4	36.7	41.3	68.5
	VFCT 1	62.2	54.9	32.3	53.4	49.9	21.2	22.8	23.3	22.6	29.6	19.7	22.3	11.3	11.9	34.8	38.1	71.2
	VFCT 2	63.1	56.0	32.5	61.4	52.1	22.7	21.5	24.0	23.0	29.7	21.2	21.6	11.2	11.5	35.8	40.0	69.7
	VFCT 3	63.1	56.0	32.5	61.4	51.9	23.5	21.4	24.3	24.0	30.7	22.1	21.7	11.2	11.4	36.9	43.4	70.5
	VFCT 4	63.0	56.2	33.1	61.8	49.9	21.0	23.4	25.8	21.8	28.5	22.3	21.5	10.6	12.4	36.3	40.9	70.7
	VFCT 5	63.2	56.0	32.4	61.3	50.4	22.5	22.3	26.6	22.3	30.7	23.3	21.5	11.0	12.1	37.7	43.1	71.3
	VFCT 6	63.0	56.1	32.3	60.4	50.5	23.5	22.2	25.7	22.3	30.9	22.6	21.9	11.4	12.4	37.7	43.4	72.0
	VFCT 7	62.7	56.0	32.3	62.2	52.3	22.5	22.6	27.5	22.9	32.3	25.8	21.9	10.8	11.8	38.8	42.5	72.7

Fig. S1

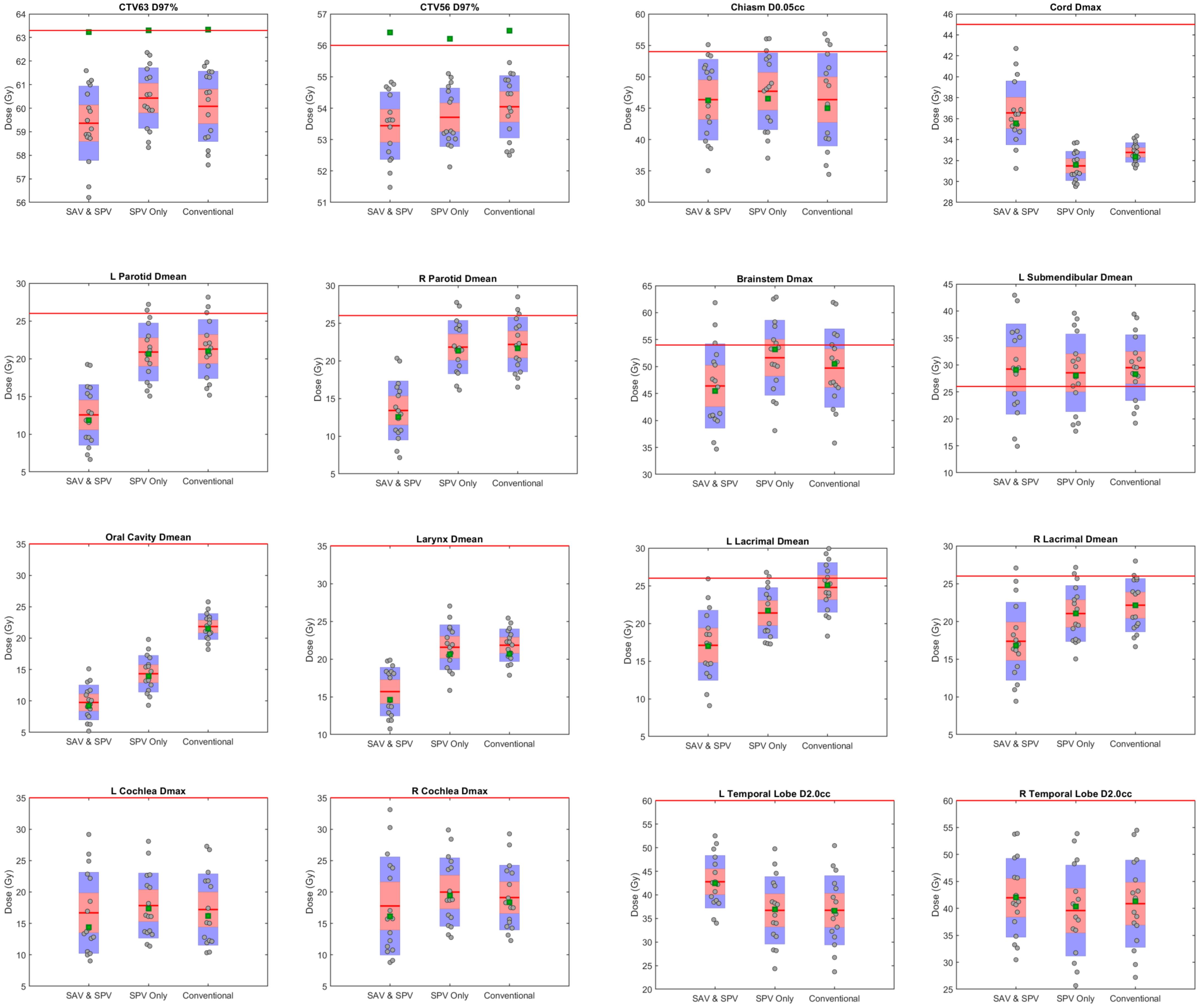
CTV63 D97%
CTV56 D97%
Chiasm D0.05cc
Cord Dmax
L Parotid Dmean
R Parotid Dmean
Brainstem Dmax
L Submendibular Dmean
Oral Cavity Dmean
Larynx Dmean
L Lacrimal Dmean
R Lacrimal Dmean
L Cochlea Dmax
R Cochlea Dmax
L Temporal Lobe D2.0cc
R Temporal Lobe D2.0cc
Dose (Gy)
SAV & SPV
SPV Only
Conventional

Fig. S2

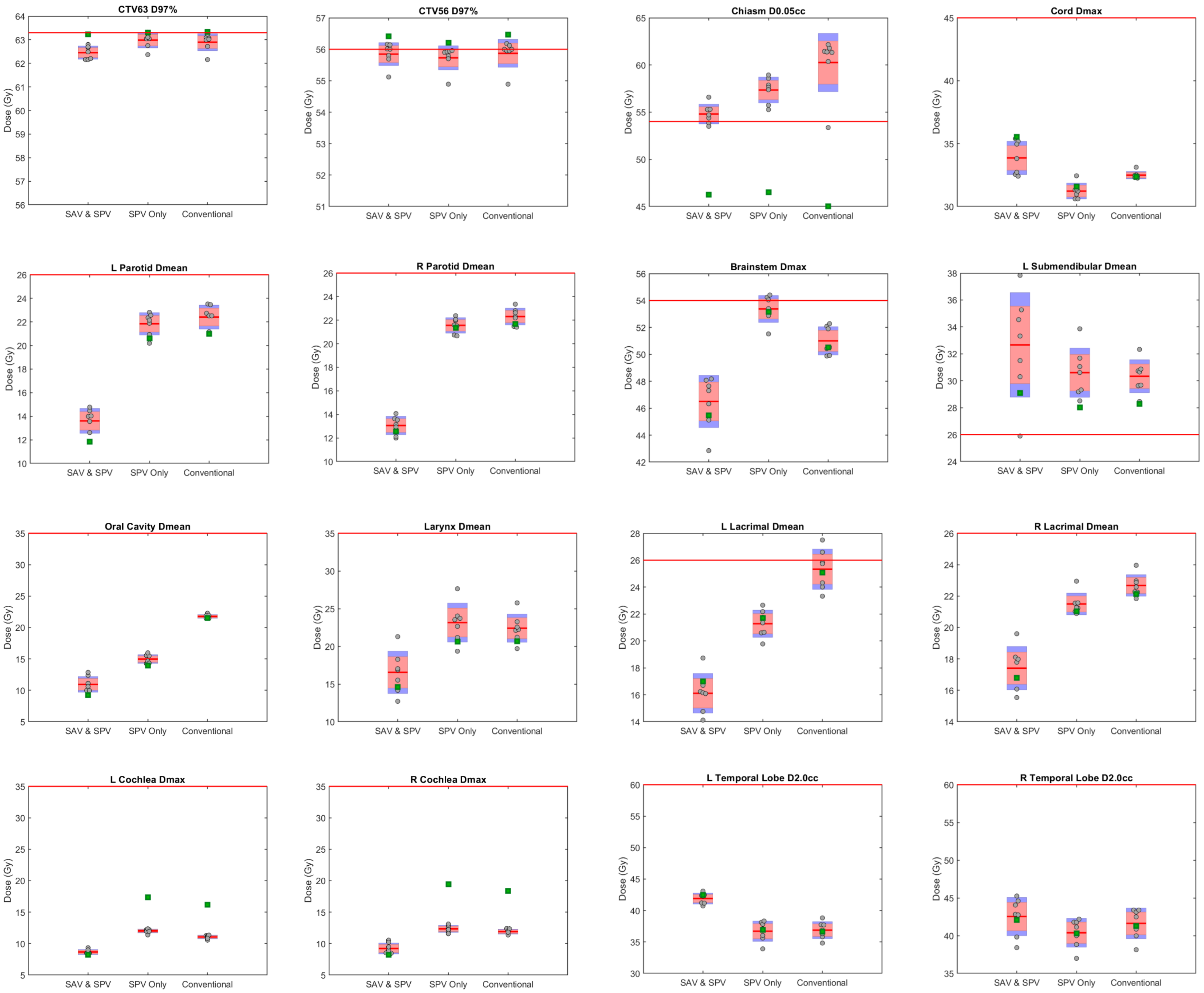

CTV63 D97%
CTV56 D97%
Chiasm D0.05cc
Cord Dmax
L Parotid Dmean
R Parotid Dmean
Brainstem Dmax
L Submendibular Dmean
Oral Cavity Dmean
Larynx Dmean
L Lacrimal Dmean
R Lacrimal Dmean
L Cochlea Dmax
R Cochlea Dmax
L Temporal Lobe D2.0cc
R Temporal Lobe D2.0cc
Dose (Gy)
SAV & SPV
SPV Only
Conventional

Figure S3:
Case 1 for
4-beam arrangement

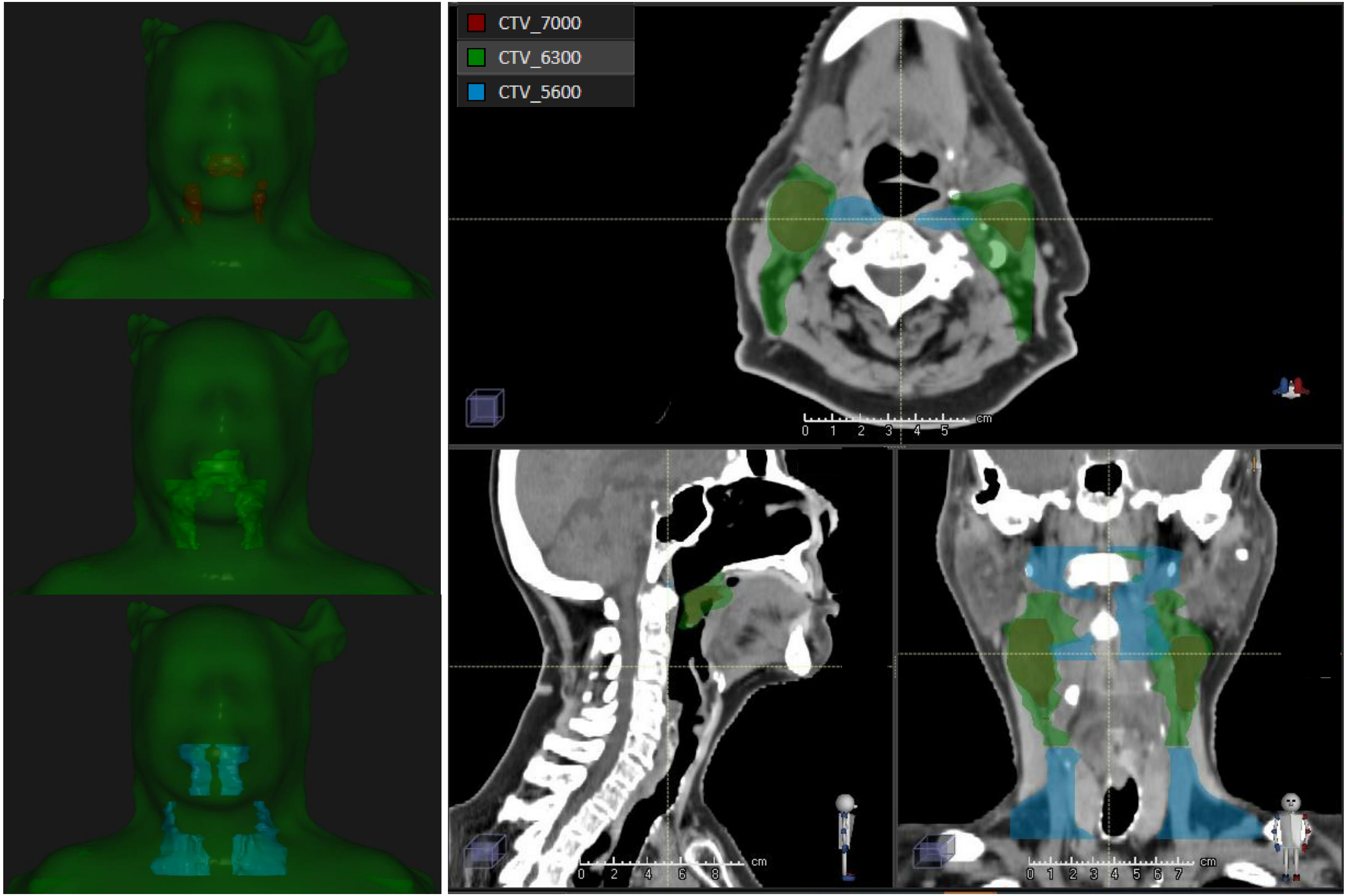

Figure S4:
Case 2 for
5-beam arrangement

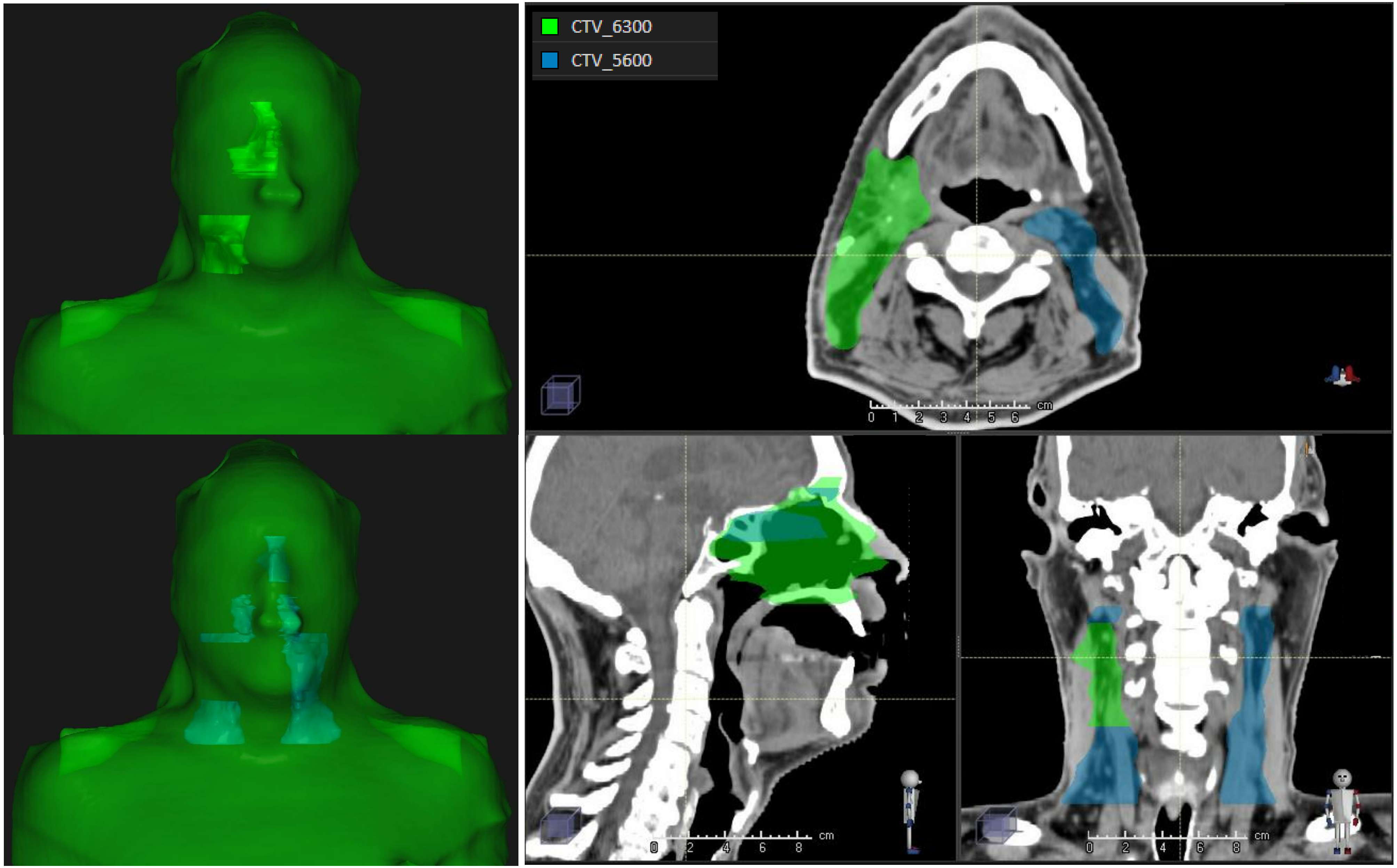

Figure S5:
Case 3 for variant 5-beam arrangement

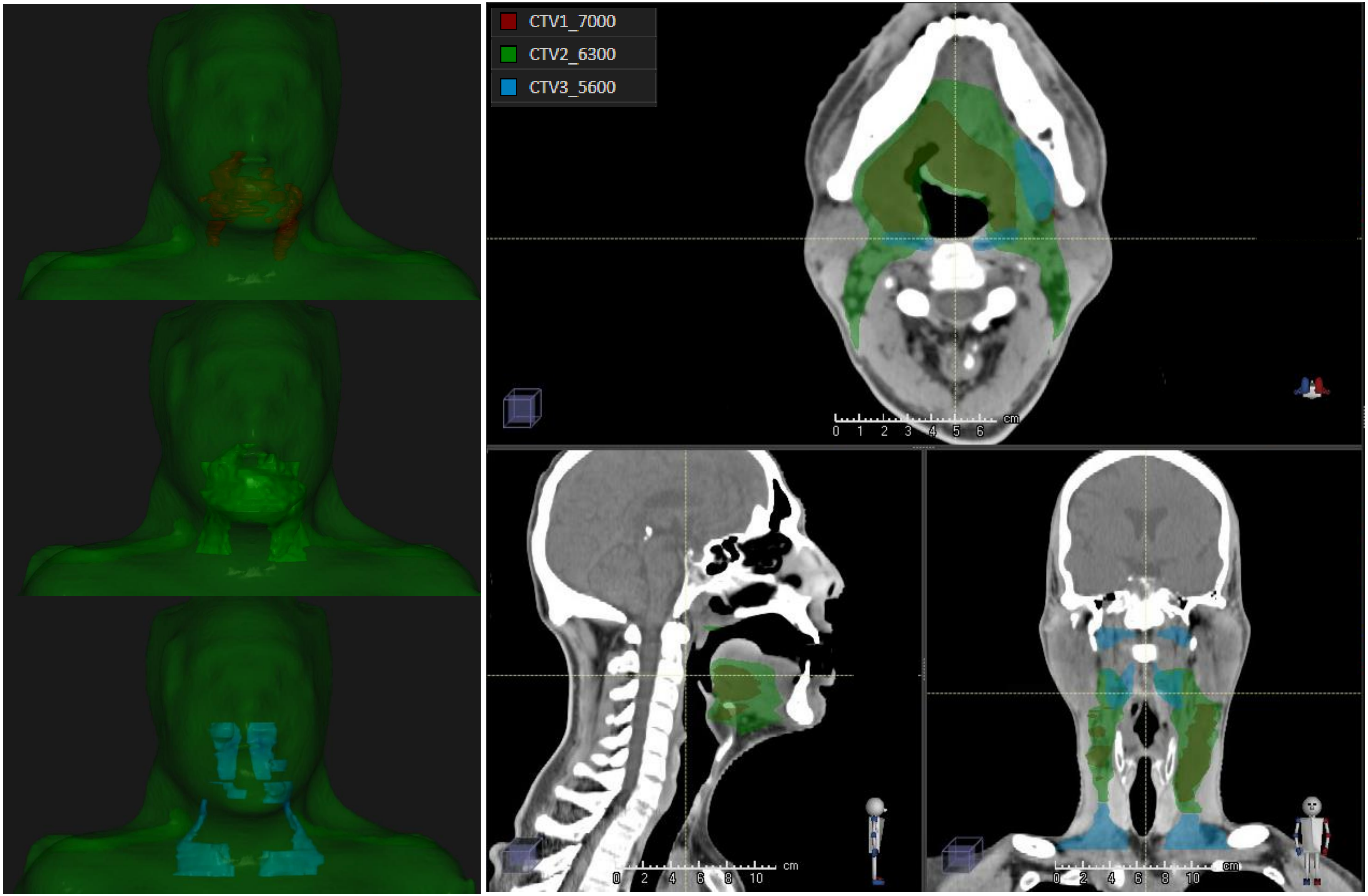

Table S1

MFOcon					*MFOspv*					*MFOspv/sav*				
**Structure**	**Objective**	**Constraint**	**Robust**	**Weight**	**Structure**	**Objective**	**Constraint**	**Robust**	**Weight**	**Structure**	**Objective**	**Constraint**	**Robust**	**Weight**
CTV 63	Min 63 Gy		Y	100	CTV 63	Min 63 Gy		Y	100	CTV 63	Min 63 Gy		Y	100
CTV 56	Min 56 Gy		Y	100	CTV 56	Min 56 Gy		Y	100	CTV 56	Min 56 Gy		Y	100
PTV 63	Uniform 63 Gy			300	PTV 63	Uniform 63 Gy			300	PTV 63	Uniform 63 Gy			300
PTV 56	Uniform 56 Gy			300	PTV 56	Uniform 56 Gy			300	PTV 56	Uniform 56 Gy			300
Parotid L	Max EUD 20 Gy			1	Parotid L	Max EUD 20 Gy			1	Parotid L	Max EUD 20 Gy			1
Parotid R	Max EUD 20 Gy			1	Parotid R	Max EUD 20 Gy			1	Parotid R	Max EUD 20 Gy			1
Larynx	Max EUD 20 Gy			10	Larynx	Max EUD 20 Gy			10	Larynx	Max EUD 20 Gy			10
Oral Cavity	Max EUD 20 Gy			1	Oral Cavity	Max EUD 20 Gy			1	Oral Cavity	Max EUD 20 Gy			1
Submend L	Max EUD 26 Gy			10	Submend L	Max EUD 26 Gy			10	Submend L	Max EUD 26 Gy			10
Lacrimal L	Max EUD 26 Gy			1	Lacrimal L	Max EUD 26 Gy			1	Lacrimal L	Max EUD 26 Gy			1
Lacrimal R	Max EUD 26 Gy			1	Lacrimal R	Max EUD 26 Gy			1	Lacrimal R	Max EUD 26 Gy			1
Cochlea L	Max 30 Gy	Y			Cochlea L	Max 30 Gy	Y			Cochlea L	Max 30 Gy	Y		
Cochlea R	Max 30Gy	Y			Cochlea R	Max 30Gy	Y			Cochlea R	Max 30Gy	Y		
Brainstem	Max 53 Gy	Y			Brainstem	Max 53 Gy	Y			Brainstem	Max 53 Gy	Y		
Cord	Max 35 Gy	Y			Cord	Max 35 Gy	Y			Cord	Max 35 Gy	Y		
Chiasm	Max 53 Gy	Y			Chiasm	Max 53 Gy	Y			Chiasm	Max 53 Gy	Y		
Optic Nrv L	Max 48 Gy	Y			Optic Nrv L	Max 48 Gy	Y			Optic Nrv L	Max 48 Gy	Y		
Optic Nrv R	Max 49 Gy	Y			Optic Nrv R	Max 49 Gy	Y			Optic Nrv R	Max 49 Gy	Y		
					SPV RPO	Max 31.5 Gy	Y			SPV RPO	Max 31.5 Gy	Y		
					SPV RAO	Max 31.5 Gy	Y			SPV RAO	Max 31.5 Gy	Y		
					SPV AP	Max 31.5 Gy	Y			SPV AP	Max 31.5 Gy	Y		
					SPV LAO	Max 31.5 Gy	Y			SPV LAO	Max 31.5 Gy	Y		
					SPV LAO	Max 31.5 Gy	Y			SPV LAO	Max 31.5 Gy	Y		
										SFO AP 56	Uniform 28 Gy			25
										SFO AP 63	Uniform 31.5 Gy			25
										SFO LPO	Uniform 28 Gy			25
										SFO RPO 56	Uniform 28 Gy			25
										SFO RPO 63	Uniform 31.5 Gy			25

Table 1 Click here to access/download;Table;Table_1_v1.docx

Table 1

		CTV63 D97%	CTV56 D97%	Cord Dmax	Chiasm D0.05cc	Brainstem Dmax	Parotid Left Dmean	Parotid Right Dmean	Lacrimal Gland Left Dmean	Lacrimal Gland Right Dmean	Submendibular Gland Left Dmean	Larynx Dmean	Oral Cavity Dmean	Cochlea Left Dmax	Cochlea Right Dmax	Temporal Lobe Left D2.0cc	Temporal Lobe Right D2.0cc	External D1.0cc
MFO using SPV and SAV	Nominal	63.2	56.4	35.6	46.3	45.5	11.8	12.6	17.0	16.8	29.1	14.6	9.2	14.4	16.1	42.5	42.1	67.4
	Scenario 1	60.6	54.4	41.2	41.0	40.8	9.6	17.0	18.6	27.1	36.0	19.7	13.0	10.5	11.3	34.8	45.8	73.2
	Scenario 2	61.6	54.7	39.5	51.8	47.7	11.8	20.3	14.7	19.2	34.5	18.4	9.1	16.9	26.1	39.6	53.8	72.7
	Scenario 3	61.0	52.6	35.9	45.4	54.4	9.6	16.5	14.6	25.3	29.4	18.1	11.5	13.4	15.7	38.5	45.7	72.9
	Scenario 4	61.1	52.4	35.3	55.1	61.9	11.6	20.0	10.6	16.3	28.3	17.5	7.8	24.9	33.1	42.6	53.9	71.7
	Scenario 5	60.0	54.6	34.9	39.7	41.0	16.3	10.8	25.9	18.2	42.9	19.9	15.1	10.0	10.5	42.8	33.2	73.1
	Scenario 6	61.2	54.8	31.2	51.4	47.4	19.2	13.9	21.1	13.2	41.9	18.3	11.0	22.9	24.2	49.7	40.9	70.6
	Scenario 7	58.9	53.6	35.4	43.6	50.9	16.2	10.5	22.1	17.5	36.3	19.1	13.3	13.7	13.5	46.5	34.8	73.6
	Scenario 8	58.9	53.6	34.0	53.5	57.7	19.1	13.4	17.4	10.9	35.1	18.1	9.5	29.2	30.3	52.5	42.4	71.9
	Scenario 9	59.1	52.3	42.7	38.9	35.9	7.3	12.9	17.2	24.1	22.7	13.8	10.2	10.2	8.8	34.0	40.7	70.1
	Scenario 10	59.5	53.4	40.2	50.7	40.2	9.2	15.9	13.4	17.0	21.1	11.9	6.3	14.2	17.0	38.8	49.4	69.6
	Scenario 11	59.9	51.5	36.8	42.8	46.2	6.7	12.3	13.0	22.0	16.3	11.9	8.7	12.6	12.3	38.2	41.2	69.7
	Scenario 12	58.8	51.9	36.4	53.3	52.2	8.2	15.4	9.1	14.0	14.9	10.8	5.2	22.2	23.8	42.2	49.7	69.3
	Scenario 13	57.7	53.6	36.4	35.0	34.7	13.0	8.0	23.4	16.2	31.0	14.5	11.7	9.0	9.1	40.7	30.5	69.7
	Scenario 14	58.7	54.8	33.0	49.8	39.9	15.4	10.8	18.5	11.6	29.5	12.9	7.5	18.5	15.8	48.0	37.5	69.1
	Scenario 15	56.7	52.9	36.9	38.6	41.3	12.8	7.2	19.4	15.7	24.7	13.7	10.1	12.8	10.8	44.8	32.6	69.2
	Scenario 16	56.2	53.9	34.7	50.9	50.3	15.0	9.7	14.7	9.4	23.1	12.5	6.3	26.0	22.2	50.9	39.3	69.7
MFO using SPV only	Nominal	63.3	56.2	31.6	46.5	53.2	20.6	21.3	21.7	21.0	28.0	20.6	13.9	17.4	19.5	36.9	40.3	67.1
	Scenario 1	62.4	54.7	30.7	43.5	45.9	16.9	24.3	19.1	26.3	37.4	25.6	18.3	11.7	16.3	28.3	40.8	71.1
	Scenario 2	62.3	54.5	30.7	54.1	53.3	20.6	27.7	20.0	25.7	36.3	22.9	13.9	18.2	25.6	35.7	52.5	70.1
	Scenario 3	61.7	53.2	32.9	47.7	55.0	16.4	24.1	19.0	27.2	30.7	22.1	15.8	13.7	18.7	31.7	43.1	70.4
	Scenario 4	61.9	52.1	32.7	56.0	62.5	19.9	27.3	19.0	24.5	29.7	19.9	11.7	22.6	29.9	38.5	53.9	69.2
	Scenario 5	60.6	54.8	30.2	41.2	43.5	23.0	18.5	26.8	17.4	39.6	27.0	19.8	13.5	13.2	38.3	29.8	70.0
	Scenario 6	61.3	55.1	29.5	52.8	54.3	27.2	22.0	25.5	19.0	38.5	24.0	15.4	22.8	20.2	46.6	40.4	69.5
	Scenario 7	60.1	53.3	32.8	48.1	53.5	22.5	18.4	26.2	19.6	32.1	24.2	17.3	16.3	14.7	42.5	31.8	69.6
	Scenario 8	60.0	52.8	30.9	56.1	62.9	26.4	21.4	24.8	19.5	31.1	21.5	13.2	28.1	24.9	49.8	41.7	68.5
	Scenario 9	61.3	53.0	31.6	41.2	43.2	15.7	21.7	17.4	22.4	26.1	21.9	15.5	11.4	16.0	24.4	36.2	69.1
	Scenario 10	60.6	53.3	32.0	52.0	50.4	19.4	25.3	18.2	22.9	24.9	18.9	11.2	16.1	23.6	31.2	48.3	68.6
	Scenario 11	59.9	52.8	33.7	43.0	50.3	15.0	21.4	17.3	23.3	18.9	18.4	13.3	13.8	18.7	28.2	38.4	68.8
	Scenario 12	59.9	53.2	33.7	53.2	57.5	18.3	24.7	17.3	21.6	17.7	15.9	9.3	21.0	28.4	34.1	49.0	68.2

	Scenario 13	59.1	54.3	29.9	37.0	38.1	21.5	16.6	23.9	15.0	27.8	23.6	16.9	13.2	12.8	34.0	25.7	68.2
	Scenario 14	59.0	55.0	29.7	48.4	50.1	25.5	20.2	22.6	17.2	26.5	20.5	12.5	20.7	18.8	41.9	36.0	68.4
	Scenario 15	58.6	53.0	32.1	39.8	47.5	21.1	16.1	23.4	17.4	20.4	20.8	14.7	16.1	14.4	38.0	28.2	67.9
	Scenario 16	58.3	54.2	30.8	49.0	58.3	24.7	19.3	21.7	17.6	19.2	18.0	10.7	26.2	23.9	44.6	37.8	67.5
Conventional MFO	Nominal	63.3	56.5	32.4	45.0	50.5	21.0	21.7	25.1	22.1	28.3	20.7	21.5	16.2	18.4	36.7	41.3	68.5
	Scenario 1	61.8	54.7	33.0	41.1	44.5	17.5	25.6	21.0	26.1	36.5	23.8	23.9	10.3	15.6	26.7	42.9	75.2
	Scenario 2	62.0	54.9	32.5	56.8	51.6	22.0	28.5	24.1	25.5	35.2	22.8	21.1	17.4	23.4	35.0	53.7	76.2
	Scenario 3	61.3	53.8	31.6	45.1	53.3	16.5	24.4	23.2	28.0	28.4	22.3	21.6	12.8	18.3	32.3	45.3	75.2
	Scenario 4	61.6	53.9	31.3	55.8	61.9	20.8	26.8	25.7	25.5	26.9	21.3	19.0	23.2	29.3	39.5	54.5	74.7
	Scenario 5	61.5	54.9	33.6	40.2	42.0	23.1	19.5	27.8	17.8	39.4	25.4	25.8	11.9	13.1	36.8	32.1	73.0
	Scenario 6	61.3	55.5	32.6	53.6	50.9	28.2	22.0	29.3	19.1	38.7	24.1	23.1	21.8	19.0	46.2	41.9	73.0
	Scenario 7	60.7	54.5	32.0	45.7	54.0	22.1	18.2	28.5	20.5	30.6	24.8	23.4	15.1	14.5	42.4	34.0	73.4
	Scenario 8	60.4	55.1	31.6	55.1	61.6	26.9	20.4	29.9	20.6	29.6	23.2	20.9	27.3	24.2	50.5	43.2	71.4
	Scenario 9	60.7	52.9	34.1	35.9	41.2	16.0	23.4	18.3	23.9	29.5	20.4	22.9	10.4	15.2	23.7	37.3	71.0
	Scenario 10	59.7	52.6	33.3	50.5	47.0	20.2	26.2	21.8	23.6	27.9	19.2	20.1	16.3	21.2	31.1	48.6	71.9
	Scenario 11	58.7	52.5	32.8	38.0	47.1	15.2	22.3	20.8	25.8	21.0	19.3	20.7	12.3	17.5	29.4	39.3	71.4
	Scenario 12	58.8	52.6	32.2	49.3	56.1	19.0	24.6	23.7	23.9	19.2	17.9	18.2	21.8	27.5	36.0	49.0	69.8
	Scenario 13	59.1	54.0	34.3	34.5	35.8	21.4	17.7	24.1	16.6	32.2	22.3	24.7	12.2	12.3	33.2	27.2	70.2
	Scenario 14	58.2	55.1	33.8	48.6	46.5	26.1	20.1	26.1	18.2	31.1	20.8	22.0	20.9	17.5	41.4	36.8	70.2
	Scenario 15	58.0	53.3	33.3	40.1	46.1	20.6	16.5	25.3	19.4	23.4	21.9	22.4	15.0	14.3	38.5	29.6	69.3
	Scenario 16	57.6	54.5	32.3	51.4	55.8	25.0	18.5	27.0	19.7	22.1	20.1	19.9	26.7	23.0	45.2	38.5	68.8

Table 2

		CTV63 D97%	CTV56 D97%	Cord Dmax	Chiasm D0.05cc	Brainstem Dmax	Parotid Left Dmean	Parotid Right Dmean	Lacrimal Gland Left Dmean	Lacrimal Gland Right Dmean	Submendibular Gland Left Dmean	Larynx Dmean	Oral Cavity Dmean	Cochlea Left Dmax	Cochlea Right Dmax	Temporal Lobe Left D2.0cc	Temporal Lobe Right D2.0cc	External D1.0cc
MFO using SPV and SAV	Nominal	63.2	56.4	35.6	46.3	45.5	11.8	12.6	17.0	16.8	29.1	14.6	9.2	8.2	8.2	42.5	42.1	67.4
	VFCT 1	62.2	55.1	33.8	53.8	48.1	12.6	13.6	16.2	19.6	25.9	12.7	11.1	9.4	8.5	40.7	38.4	69.4
	VFCT 2	62.8	56.2	32.6	55.3	46.3	14.0	12.0	16.2	17.8	30.3	14.2	9.7	8.5	8.5	41.2	39.8	68.0
	VFCT 3	62.7	56.1	32.4	55.3	47.3	14.5	12.1	14.1	18.1	33.3	15.5	10.0	8.3	8.5	42.3	42.8	68.3
	VFCT 4	62.2	56.0	35.4	53.5	42.9	11.8	13.5	18.7	18.0	31.5	16.9	10.6	8.3	8.9	41.2	42.8	68.3
	VFCT 5	62.7	56.0	35.2	54.4	47.7	13.6	13.1	16.7	16.8	34.5	17.0	10.0	8.5	9.5	42.4	44.1	69.5
	VFCT 6	62.5	55.8	32.7	54.7	45.1	14.0	12.9	16.1	16.1	35.3	18.3	12.4	8.8	10.6	42.5	45.3	69.4
	VFCT 7	62.2	55.7	35.0	56.6	48.2	14.8	14.1	14.8	15.5	37.8	21.3	12.9	9.0	10.2	43.1	44.6	70.6
MFO using SPV only	Nominal	63.3	56.2	31.6	46.5	53.2	20.6	21.3	21.7	21.0	28.0	20.6	13.9	17.4	19.5	36.9	40.3	67.1
	VFCT 1	62.4	54.9	31.0	55.7	52.9	20.9	22.0	20.6	21.0	29.2	19.4	15.6	12.0	12.2	33.9	37.0	68.1
	VFCT 2	63.0	55.9	30.6	58.6	54.2	22.4	20.7	20.6	21.6	29.3	21.2	14.2	12.2	11.9	35.6	38.8	67.8
	VFCT 3	63.3	55.9	30.6	58.9	54.1	22.8	20.7	19.8	23.0	30.6	22.7	14.6	12.2	11.6	37.1	41.8	67.8
	VFCT 4	63.1	55.9	31.3	55.3	53.1	20.2	22.4	22.7	20.9	28.5	23.5	14.7	11.4	13.0	36.0	40.0	68.7
	VFCT 5	63.3	56.0	31.2	57.9	51.5	21.9	21.6	22.2	21.2	31.1	24.0	14.3	12.0	12.5	38.1	41.8	68.3
	VFCT 6	63.1	55.8	31.4	57.6	53.4	22.6	21.5	21.4	21.3	31.7	23.7	15.5	12.4	13.1	37.8	42.2	69.2
	VFCT 7	62.8	55.7	32.4	57.4	54.4	22.2	22.1	21.8	21.6	33.9	27.6	16.0	11.9	12.2	38.3	41.1	69.0
Conventional MFO	Nominal	63.3	56.5	32.4	45.0	50.5	21.0	21.7	25.1	22.1	28.3	20.7	21.5	16.2	18.4	36.7	41.3	68.5
	VFCT 1	62.2	54.9	32.3	53.4	49.9	21.2	22.8	23.3	22.6	29.6	19.7	22.3	11.3	11.9	34.8	38.1	71.2
	VFCT 2	63.1	56.0	32.5	61.4	52.1	22.7	21.5	24.0	23.0	29.7	21.2	21.6	11.2	11.5	35.8	40.0	69.7
	VFCT 3	63.1	56.0	32.5	61.4	51.9	23.5	21.4	24.3	24.0	30.7	22.1	21.7	11.2	11.4	36.9	43.4	70.5
	VFCT 4	63.0	56.2	33.1	61.8	49.9	21.0	23.4	25.8	21.8	28.5	22.3	21.5	10.6	12.4	36.3	40.9	70.7
	VFCT 5	63.2	56.0	32.4	61.3	50.4	22.5	22.3	26.6	22.3	30.7	23.3	21.5	11.0	12.1	37.7	43.1	71.3
	VFCT 6	63.0	56.1	32.3	60.4	50.5	23.5	22.2	25.7	22.3	30.9	22.6	21.9	11.4	12.4	37.7	43.4	72.0
	VFCT 7	62.7	56.0	32.3	62.2	52.3	22.5	22.6	27.5	22.9	32.3	25.8	21.9	10.8	11.8	38.8	42.5	72.7

Supplemental Material
Click here to access/download;Supplemental Material;Supplemental_R1_v1.docx

Supplemental Information:

Method for sinus involvement using the 5-beam technique

For the sinus region, an SFO structure is created for the AP beam excluding regions of the PTV that are posterior to the eyes. The AP beam is used to deliver half of the prescription uniformly to its SFO structure, and the remaining dose is contributed in MFO dose distributions by the other four obliques. The SAV in the sinus region includes the eyes, brainstem, and cochlea. The AP beam is typically tilted by about +/- 3 degrees to prevent the beam from being tangent to the bone and air interface in the sinus. For cases where the target volume extends to spaces behind the eyes, the eye and temporal lobe doses can become a concern, and a sixth non-coplanar beam from the superior anterior direction can be used to reduce the eye and temporal lobe dose.

Discussion on inter-field robustness optimization settings

Given the currently available computational resources in our clinics, one iteration of optimization for a 4-beam H&N case using inter-field robustness optimization will take about one to two day(s), using a simple robustness setting of 7 isocenter locations (original, sup/inf, ant/post, and left/right) and 3 range uncertainty values (original, over- and under-range). Since the number of robustness scenarios grows exponentially with the number of beams, a 5-beam H&N case using inter-field robustness, even with a reduced robustness setting, i.e. 5 isocenter locations (original, ant/post, and left/right) and 3 range uncertainty values (original, over- and under-range), has significantly more scenarios and this calculation can take two to three days for a single optimization. A typical bi-lateral H&N plan needs about 5 to 8 optimization iterations to complete. This prolonged planning time has effectively prevented the use of inter-field robustness optimization for actual clinical use. This is also part of the reason we developed the MFOspv/sav technique to specifically guide the optimizer to deliver the desired OAR sparing with robustness against inter-fractional variations in VFCTs to reduce frequent re-plans. We suspect for un-guided MFO planning techniques, such as MFOcon, inter-field robustness optimization will be required in order to achieve consistent inter-fractional robustness in VFCTs. Since the implementation of our current technique, i.e. the MFOspv/sav with sync-field robustness optimization, we have indeed seen a reduction in the amount of optimization time as well as less of a need for re-plans over the treatment course.

Supplemental figure and table captions

Figure S1: Robustness evaluation of the three MFO planning techniques using simulated scenarios. Target and OAR dose statistics are from Table 1. The mean (red horizontal line), one and two standard deviations (pink and purple bars), are shown with all 16 scenarios (grey circles). The nominal plans' dose values are also shown (green squares). The red horizontal line is the dose criteria.

Figure S2: Inter-fraction plan robustness evaluation of the three MFO planning techniques using VFCTs taken weekly over the treatment course. Target and OAR dose statistics are from Table 2. Same color legends as Fig. S1.

Figure S3: Target contours for the case. This case is used to demonstrate the SPV/SAV planning technique's 4-beam arrangement. See text and Fig. 1 for SPVs, SAVs and structures for SFO-like dose.

Figure S4: Target contours for the case. This case is used to demonstrate the SPV/SAV planning technique's 5-beam arrangement. See text and Fig. 2(a) for beam arrangements, SPVs and SAVs.

Figure S5: Target contours for the case. This case is used to demonstrate the SPV/SAV planning technique's variant 5-beam arrangement. See text and Fig. 2(b, c, d) for SPVs, SAVs and structures for SFO-like dose.

Table S1: Optimization objectives and associated parameters for the three MFO plans, i.e. MFOcon, MFOspv and MFOspv/sav. These three MFO plans were forward-calculated on weekly VFCTs to evaluate the robustness of these planning techniques.

Table S1 Click here to access/download;Table;Table_S1_AllObjectives.docx

Table S1

MFOcon				
**Structure**	**Objective**	**Constraint**	**Robust**	**Weight**
CTV 63	Min 63 Gy		Y	100
CTV 56	Min 56 Gy		Y	100
PTV 63	Uniform 63 Gy			300
PTV 56	Uniform 56 Gy			300
Parotid L	Max EUD 20 Gy			1
Parotid R	Max EUD 20 Gy			1
Larynx	Max EUD 20 Gy			10
Oral Cavity	Max EUD 20 Gy			1
Submend L	Max EUD 26 Gy			10
Lacrimal L	Max EUD 26 Gy			1
Lacrimal R	Max EUD 26 Gy			1
Cochlea L	Max 30 Gy	Y		
Cochlea R	Max 30Gy	Y		
Brainstem	Max 53 Gy	Y		
Cord	Max 35 Gy	Y		
Chiasm	Max 53 Gy	Y		
Optic Nrv L	Max 48 Gy	Y		
Optic Nrv R	Max 49 Gy	Y		

MFOspv				
**Structure**	**Objective**	**Constraint**	**Robust**	**Weight**
CTV 63	Min 63 Gy		Y	100
CTV 56	Min 56 Gy		Y	100
PTV 63	Uniform 63 Gy			300
PTV 56	Uniform 56 Gy			300
Parotid L	Max EUD 20 Gy			1
Parotid R	Max EUD 20 Gy			1
Larynx	Max EUD 20 Gy			10
Oral Cavity	Max EUD 20 Gy			1
Submend L	Max EUD 26 Gy			10
Lacrimal L	Max EUD 26 Gy			1
Lacrimal R	Max EUD 26 Gy			1
Cochlea L	Max 30 Gy	Y		
Cochlea R	Max 30Gy	Y		
Brainstem	Max 53 Gy	Y		
Cord	Max 35 Gy	Y		
Chiasm	Max 53 Gy	Y		
Optic Nrv L	Max 48 Gy	Y		
Optic Nrv R	Max 49 Gy	Y		
SPV RPO	Max 31.5 Gy	Y		
SPV RAO	Max 31.5 Gy	Y		
SPV AP	Max 31.5 Gy	Y		
SPV LAO	Max 31.5 Gy	Y		
SPV LAO	Max 31.5 Gy	Y		

MFOspv/sav				
**Structure**	**Objective**	**Constraint**	**Robust**	**Weight**
CTV 63	Min 63 Gy		Y	100
CTV 56	Min 56 Gy		Y	100
PTV 63	Uniform 63 Gy			300
PTV 56	Uniform 56 Gy			300
Parotid L	Max EUD 20 Gy			1
Parotid R	Max EUD 20 Gy			1
Larynx	Max EUD 20 Gy			10
Oral Cavity	Max EUD 20 Gy			1
Submend L	Max EUD 26 Gy			10
Lacrimal L	Max EUD 26 Gy			1
Lacrimal R	Max EUD 26 Gy			1
Cochlea L	Max 30 Gy	Y		
Cochlea R	Max 30Gy	Y		
Brainstem	Max 53 Gy	Y		
Cord	Max 35 Gy	Y		
Chiasm	Max 53 Gy	Y		
Optic Nrv L	Max 48 Gy	Y		
Optic Nrv R	Max 49 Gy	Y		
SPV RPO	Max 31.5 Gy	Y		
SPV RAO	Max 31.5 Gy	Y		
SPV AP	Max 31.5 Gy	Y		
SPV LAO	Max 31.5 Gy	Y		
SPV LAO	Max 31.5 Gy	Y		
SFO AP 56	Uniform 28 Gy			25
SFO AP 63	Uniform 31.5 Gy			25
SFO LPO	Uniform 28 Gy			25
SFO RPO 56	Uniform 28 Gy			25
SFO RPO 63	Uniform 31.5 Gy			25
